# Supplementary material for: Conformational plasticity across phylogenetic clusters of RND multidrug efflux pumps and its impact on substrate specificity
Source: Nat Commun. 2025 Nov 26;16:11649. doi: 10.1038/s41467-025-66751-3 (PMC12749989; doi:10.1038/s41467-025-66751-3)
Supplement: Supplementary file 1 — Supplementary Information [file 41467_2025_66751_MOESM1_ESM.pdf]

**Supplementary Information**

# Conformational plasticity across phylogenetic clusters of RND multidrug efflux pumps and its impact on substrate specificity

Mariya Lazarova[1], Thomas Eicher[1], Clara Börnsen[2], Hui Zeng[1], Mohd Athar[3], Ui Okada[4], Eiki Yamashita[5], Inga M. Spannaus[1], Max Borgosch[1], Hi-jea Cha[1], Attilio V. Vargiu[3], Satoshi Murakami[4*], Kay Diederichs[6*], Achilleas S. Frangakis[2*], Klaas M. Pos [1*]

[1] Institute of Biochemistry, Goethe-University Frankfurt, Germany

[2] Buchmann Institute for Molecular Life Sciences and Institute of Biophysics, Goethe-University Frankfurt, Germany

[3] Department of Physics, University of Cagliari, Italy

[4] Department of Life Science and Technology, Tokyo Institute of Technology, Yokohama, Japan

[5] Institute for Protein Research, Osaka University, Japan

[6] Department of Biology, University of Konstanz, Germany

** Corresponding authors*

Klaas M. Pos, pos@em.uni-frankfurt.de

Achilleas S. Frangakis, achilleas.frangakis@biophysik.org

Kay Diederichs, kay.diederichs@uni-konstanz.de

Satoshi Murakami, murakami@bio.titech.ac.jp

**This supplementary information file contains:**

**Supplementary Figures 1-26**
**Supplementary Tables 1-10**
**Supplementary Notes**
**Supplementary Methods**
**Supplementary References**

## Supplementary Figures

# Conformational plasticity across phylogenetic clusters of RND multidrug efflux pumps and its impact on substrate specificity

Mariya Lazarova[1], Thomas Eicher[1], Clara Börnsen[2], Hui Zeng[1], Mohd Athar[3], Ui Okada[4], Eiki Yamashita[5], Inga M. Spannaus[1], Max Borgosch[1], Hi-jea Cha[1], Attilio V. Vargiu[3], Satoshi Murakami[4*], Kay Diederichs[6*], Achilleas S. Frangakis[2*], Klaas M. Pos [1*]

[1] Institute of Biochemistry, Goethe-University Frankfurt, Germany

[2] Buchmann Institute for Molecular Life Sciences and Institute of Biophysics, Goethe-University Frankfurt, Germany

[3] Department of Physics, University of Cagliari, Italy

[4] Department of Life Science and Technology, Tokyo Institute of Technology, Yokohama, Japan

[5] Institute for Protein Research, Osaka University, Japan

[6] Department of Biology, University of Konstanz, Germany

** Corresponding authors*

Klaas M. Pos, pos@em.uni-frankfurt.de

Achilleas S. Frangakis, achilleas.frangakis@biophysik.org

Kay Diederichs, kay.diederichs@uni-konstanz.de

Satoshi Murakami, murakami@bio.titech.ac.jp

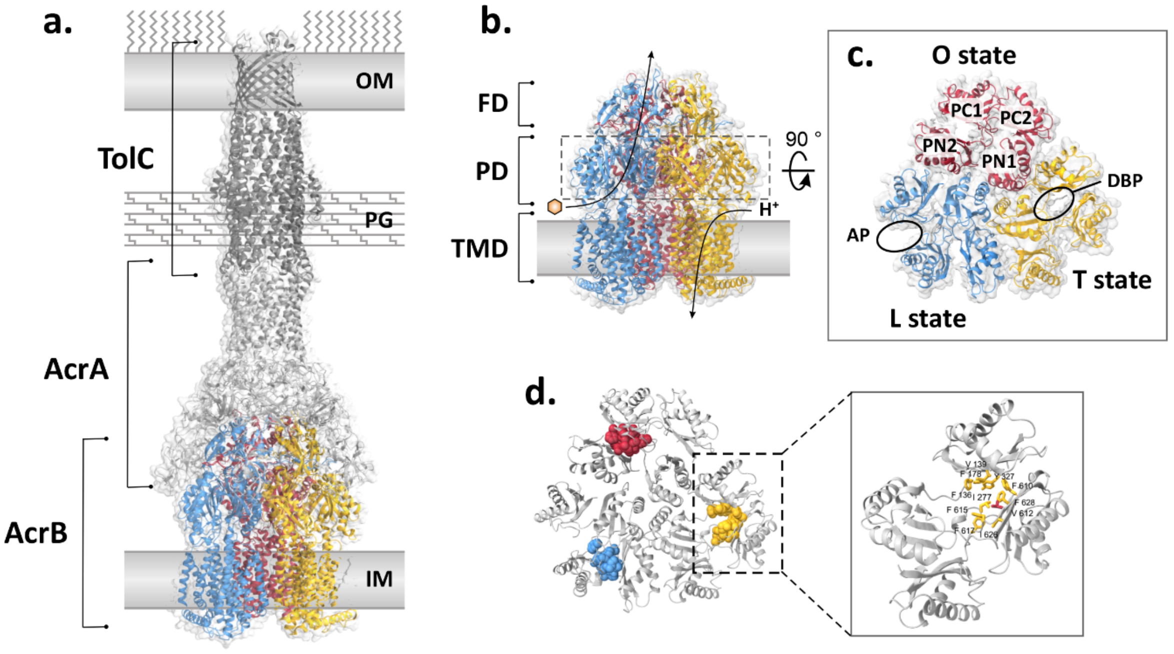


**Supplementary Figure 1: The AcrAB-TolC system from *E. coli*.** (**a.**) AcrAB-TolC is a constitutively expressed tripartite multidrug efflux system from *E. coli*. It consists of the inner membrane Resistance Nodulation and cell Division (RND) transporter AcrB, the pore forming outer membrane factor TolC and the periplasmic adaptor AcrA. (**b.**) AcrB is a drug/proton antiporter that energises the drug export and the determinant of the substrate specificity of the entire tripartite system. It forms a homotrimer in the inner membrane and each monomer can adopt one of three distinct conformational states called loose (L), tight (T) and open (O). During the catalytic transport cycle every protomer cycles through these three states in a concerted fashion with the other neighbouring protomers (LTO -> TOL -> OLT -> LTO). AcrB can be subdivided into a transmembrane domain (TMD) that contains a proton translocation network, a porter domain (PD) that contains the substrate binding pockets, and a funnel domain (FD) from where drug substrates are entering into the AcrA-TolC channel. (**c.**) The porter domain can be divided into four subdomains (PN1, PN2, PC1 and PC2). During the transport cycle, these domains move as rigid body units relative to each other thus inducing the opening and closing of the substrate binding pockets. Substrates are bound in the access pocket (AP) in the L state and in the deep binding pocket (DBP) in the T state. In the O state these pockets are closed, but an exit channel leading to the AcrA-TolC funnel is opened. (**d.**) The DBP is lined by hydrophobic, mostly aromatic, residues that form an open pocket cleft in the T state. In the O and L states this pocket is closed, and a close packing of the DPB residues is observed. Colours in all subfigures: blue – L state, yellow – T state, red – O state. PDB ID AcrAB-TolC structure: 5o66 (a.). PDB ID AcrB structure: 4dx5 (b.-d.)

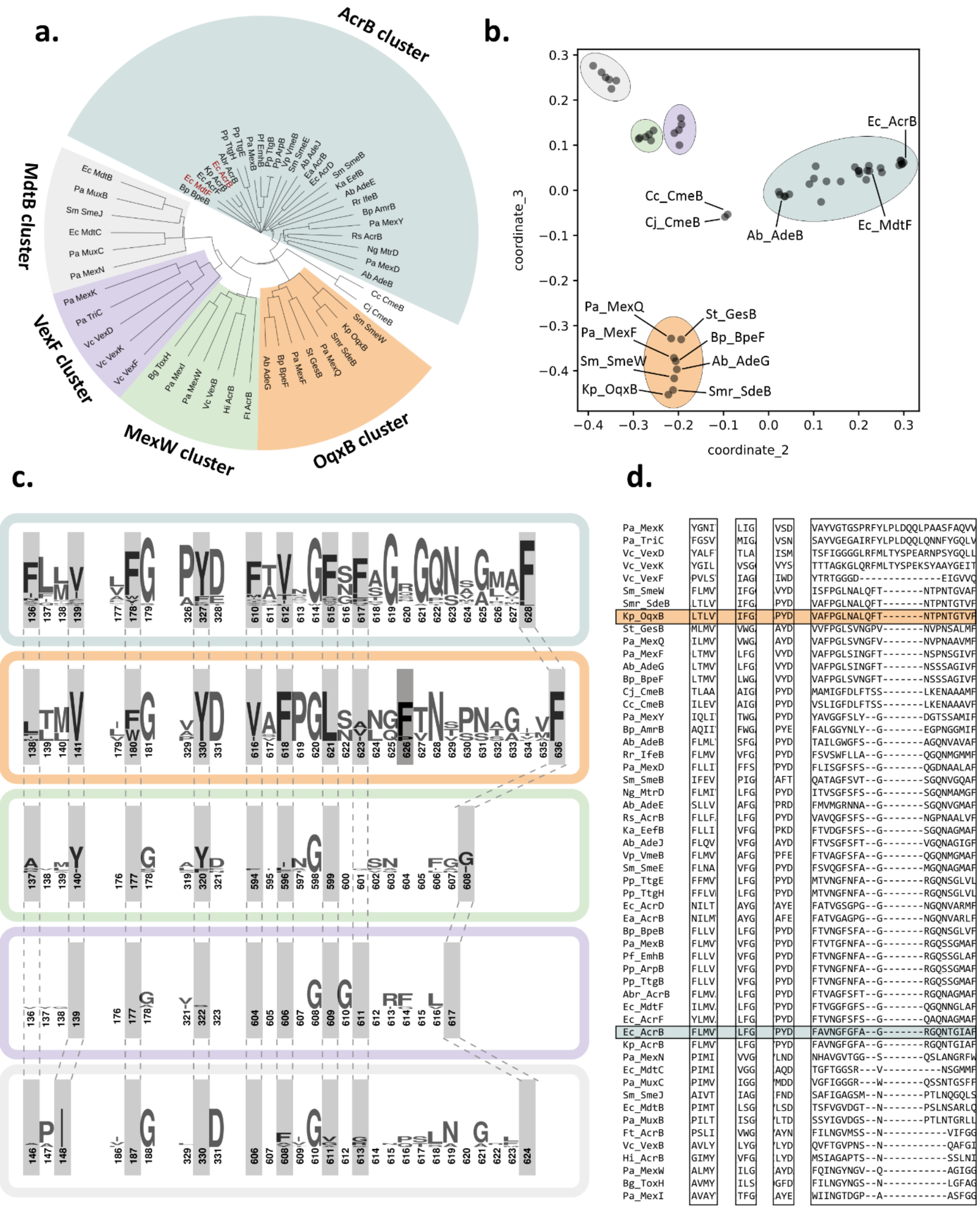


**Supplementary Figure 2: Conservation of deep binding pocket (DBP) residues in RND proteins from Gram-negative bacteria.** A phylogenetic tree (**a.**) and a cc-analysis map of pairwise sequence alignment (**b.**) of the same set of HAE-1 RND amino acid sequences (Supplementary Table 1). With both methods the same five clusters were identified (highlighted by the same colour in all subfigures). The CmeB genes did not cluster with any of the other genes. The AcrB cluster (cyan) comprises several subclusters such as the MdtF and AdeB subclusters. (**c.**) Consensus sequence of the DBP residues for each cluster. The residues numbering corresponds to the sequences of following cluster members: AcrB (cyan), OqxB (orange), MexW (green), VexF (purple) and MdtB (grey). DBP residues are highlighted in grey and dashed lines connect the equivalent residues in each consensus sequence. The

sequence alignment for the same four regions for all tested sequence is given in (d.) with AcrB and OqxB highlighted. The DBP residues are conserved in the AcrB and OqxB clusters (cyan and orange, respectively), but not in the remaining three clusters. The position equivalent to AcrB_F617 is not conserved in the OqxB cluster but is compensated by F626 (darker grey) in the latter. Abbreviations: Ec: *Escherichia coli*; Rr: *Rhizobium radiobacter*; Ab: *Acinetobacter baumannii*; Ng: *Neisseria gonorrhoeae*; Pa: *Pseudomonas aeruginosa*; Pp: *Pseudomonas putida*; Sm: *Stenotrophomonas maltophilia*; Ka: *Klebsiella aerogenes*; Bg: *Burkholderia glumae*; Cj: *Campylobacter jejuni*; Cc: *Campylobacter coli*; Bp: *Burkholderia pseudomallei*; St: *Salmonella typhimurium*; Vp: *Vibrio parahaemolyticus*; Ft: *Francisella tularensis*; Vc: *Vibrio cholerae*; Smr: *Serratia marcescens*; Rs: *Ralstonia solanacearum*; Abr: *Alcanivorax borkumensis*; Kp: *Klebsiella pneumoniae*; Ea: *Erwinia amylovora*; Hi: *Haemophilus influenzae*; Pf: *Pseudomonas fluorescens*

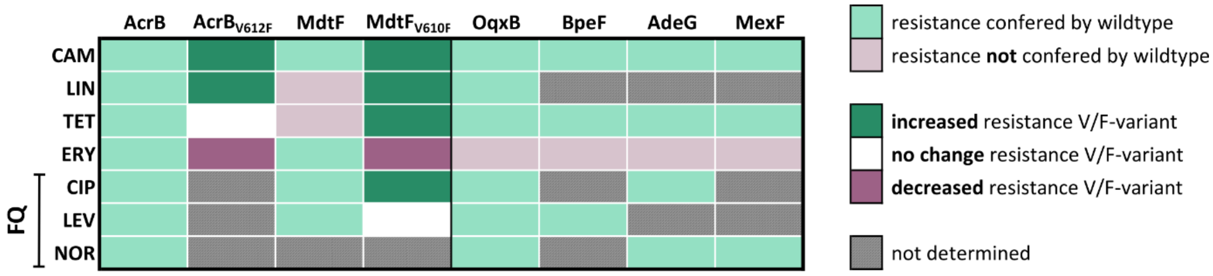


**Supplementary Figure 3: Phenotype of AcrB, MdtF and OqxB cluster representatives.** The figure shows schematically the resistance phenotype of AcrB (this study and [1–3]), MdtF [4] and several representatives of the OqxB cluster [5–9] against a panel of antibiotics. The resistance of cells expressing the respective wildtype RND pump is depicted in comparison to either cells lacking expression of this pump, or cells expressing its inactive variant. The resistance of the V/F variants of AcrB and MdtF is shown in comparison to cell expressing the respective wildtype pump. Grey values indicate not determined. Abbreviations: CAM – chloramphenicol, LIN – linezolid, TET – tetracycline, TIG – tigecycline, MIN – minocycline, ERY – erythromycin, NOV – novobiocin, CIP – ciprofloxacin, LEV – levofloxacin, NOR – norfloxacin, FQ – fluoroquinolones.

**a.**

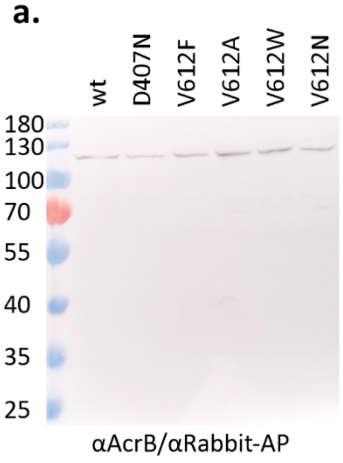


**b.**

| | wt | V612F | V612W | V612N | V612A | D407N |
|---|---|---|---|---|---|---|
| CAM | 1,00 | 2,00 | 1,67 | 1,67 | 1,67 | 0,34 |
| TIA | 1,00 | 2,00 | 1,67 | 2,00 | 1,00 | 0,50 |
| LIN | 1,00 | 1,80 | 2,00 | 1,33 | 1,83 | 0,06 |
| TET | 1,00 | 1,00 | 1,00 | 1,00 | 1,33 | 0,36 |
| OxTET | 1,00 | 2,00 | 2,00 | 2,00 | 2,00 | 0,50 |
| TIG | 1,00 | 0,82 | 1,09 | 1,00 | 1,09 | 0,55 |
| CITET | 1,00 | 1,00 | 1,00 | 1,00 | 1,00 | 0,25 |
| DXC | 1,00 | 1,00 | 1,00 | 1,00 | 1,00 | 0,15 |
| MIN | 1,00 | 0,50 | 0,72 | 0,89 | 0,72 | 0,31 |
| DIC | 1,00 | 0,50 | 0,50 | 0,50 | 1,00 | 0,07 |
| OXA | 1,00 | 0,50 | 0,67 | 0,58 | 0,83 | 0,02 |
| PIP | 1,00 | 0,31 | 0,67 | 0,58 | 0,83 | 0,22 |
| SDS* | | | | | | |
| ERY | 1,00 | 0,44 | 0,50 | 0,50 | 0,75 | 0,06 |
| CLA | 1,00 | 0,50 | 0,92 | 0,50 | 1,00 | 0,11 |
| NOV | 1,00 | 0,45 | 0,64 | 0,72 | 1,56 | 0,18 |
| FUA | 1,00 | 0,45 | 0,75 | 0,50 | 1,00 | 0,11 |
| H33342 | 1,00 | 0,34 | 0,38 | 0,83 | 0,75 | 0,20 |
| BER | 1,00 | 0,13 | n.d. | n.d | n.d. | 0,06 |

MIC variant/MIC wt (scale: 2 1 0)

**Supplementary Figure 4: Phenotype characterisation of AcrB_V612 variants.** For the functional characterisation of AcrB V612 variants, BW25113 Δ*acrB* cells were transformed with a pET24 vector encoding the AcrB variants. For each functional assay, Western blot samples were prepared from membranes isolated from the used clones to verify the presence of the AcrB variants. Western blot membranes were stained with a rabbit anti-AcrB primary antibody (Custom anti-AcrB antibody by Neosystems, France, 1:10´000) and an anti-rabbit-alkaline phosphatase conjugated secondary antibody (Anti-rabbit IgG-Alkaline phosphatase antibody, Sigma-Aldrich, Catalog # A3687-1ML, 1:1´500) [10]. A representative blot is shown in (**a**.). For all variants a band for the AcrB monomer (calculated molecular weight of 114 kDa) is visible. (**b.**) Minimal inhibitory concentration (MIC) assay. Bacterial cultures were exposed to a serial dilution of a toxic substrate. The inactive AcrB variant D407N [11] was used as negative control. The first dilution step at which no growth was detected was determined (MIC) and normalised to the wildtype (wt) MIC. Values higher than one (indicated with green colour) represent a higher MIC and values lower than one (purple) represent a lower MIC compared to the wt for each given toxin. The figure represents summary data of at least three biological replicates. The mean MIC values used for the figure generation are summarized in Supplementary Table 3 and the individual MIC values of all replicates are given in the source data. *For SDS, the MIC value of the wt exceeded the maximal tested concentration, therefore MIC values of the cells harbouring AcrB variants could not be normalised to the wt. Nonetheless, a clear reduction of the resistance against SDS was detected for cells harbouring the V612F and V612W variants (Supplementary Table 3). Abbreviations of the tested drugs, given at the left side of the figure, are specified in Supplementary Table 2. n.d.: not determined. Source data are provided as a Source Data file.

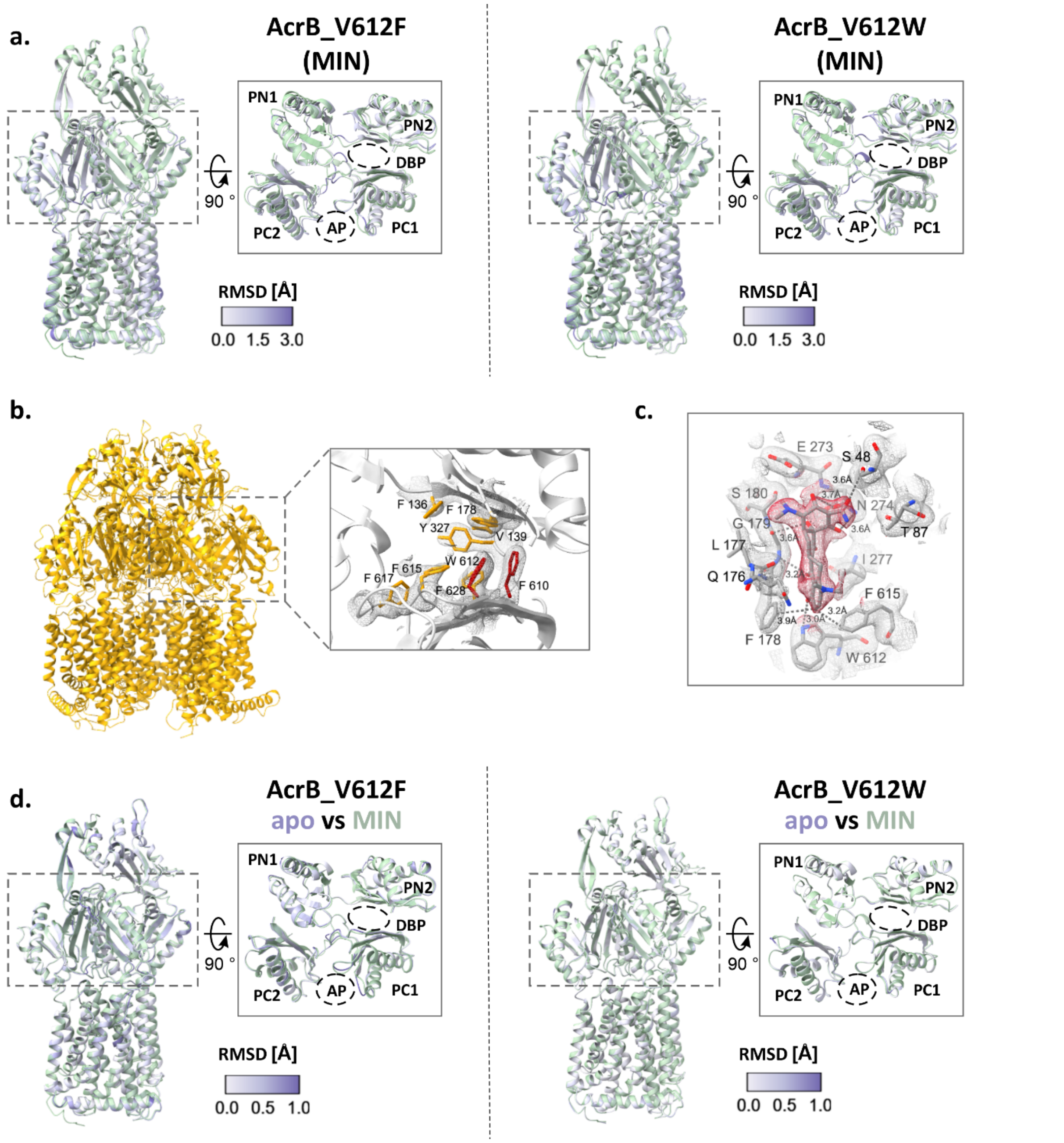


**Supplementary Figure 5: Comparison of V612F/W structures with AcrB wildtype.** (**a.**) The V612F/W structures with bound minocycline were aligned with the AcrB wildtype structure in the T state (PDB ID: 4dx5, green) and coloured by the RMSD values as indicated in the colour key. Inset: top view of the porter domain. The PN1, PN2, PC1 and PC2 subdomains are indicated. AP: Access Pocket, DBP: Deep Binding Pocket, MIN: minocycline. (**b.**) Crystallographic structure of AcrB V612W in the TTT state. The deep binding pocket residues in AcrB_V612W are shown in the inlet on the right. The crystallographic $2F_o$-$F_c$ map is shown at $\sigma$1 (mesh). (**c.**) Minocycline interactions in the deep binding pocket of AcrB V612W. The crystallographic $2F_o$-$F_c$ map is shown at $\sigma$1 (mesh) and the densities for minocycline (shown as sticks) are highlighted in red. Residues with at least one atom within 4 Å distance of minocycline are shown as sticks and indicated with single letter amino acid code and position number. The distances between the side chains and minocycline (dashed lines) are indicated. Carbon atoms are given in grey, oxygen in red, and nitrogen in blue. (**d.**) The V612F/W structures with minocycline (green) were aligned with the respective V612F/W structure in the apo state (coloured by the RMSD as indicated in the colour key). Annotation as in (a).

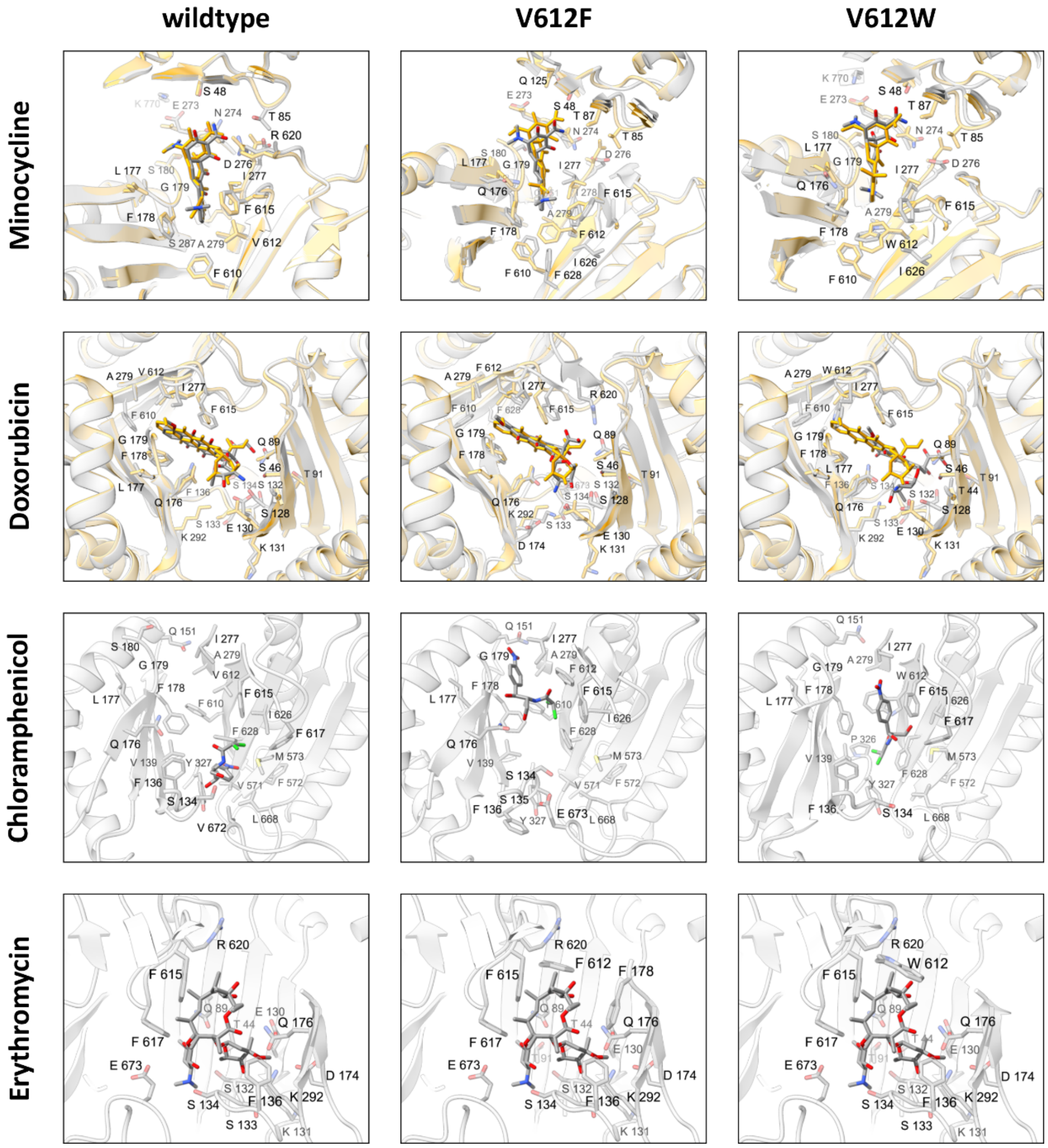


**Supplementary Figure 6: Docking of AcrB substrates in the deep binding pocket of AcrB wildtype and the V612F/W variants.** The substrates and potential interacting residues (< 4 Å) are shown as sticks. All docking poses are coloured by atom type with carbon atoms in grey, oxygen in red, and nitrogen in blue. For minocycline, the docking poses were overlayed with the crystallographic structures of the wildtype (PDB ID: 4dx5) or the crystallographic structures of the respective V612 variant (this study, yellow). For doxorubicin, all docking poses were overlayed with the crystallographic structure of the AcrB wildtype (PDB ID: 4dx7, yellow). For a detailed discussion of the results see the Supplementary Notes below.

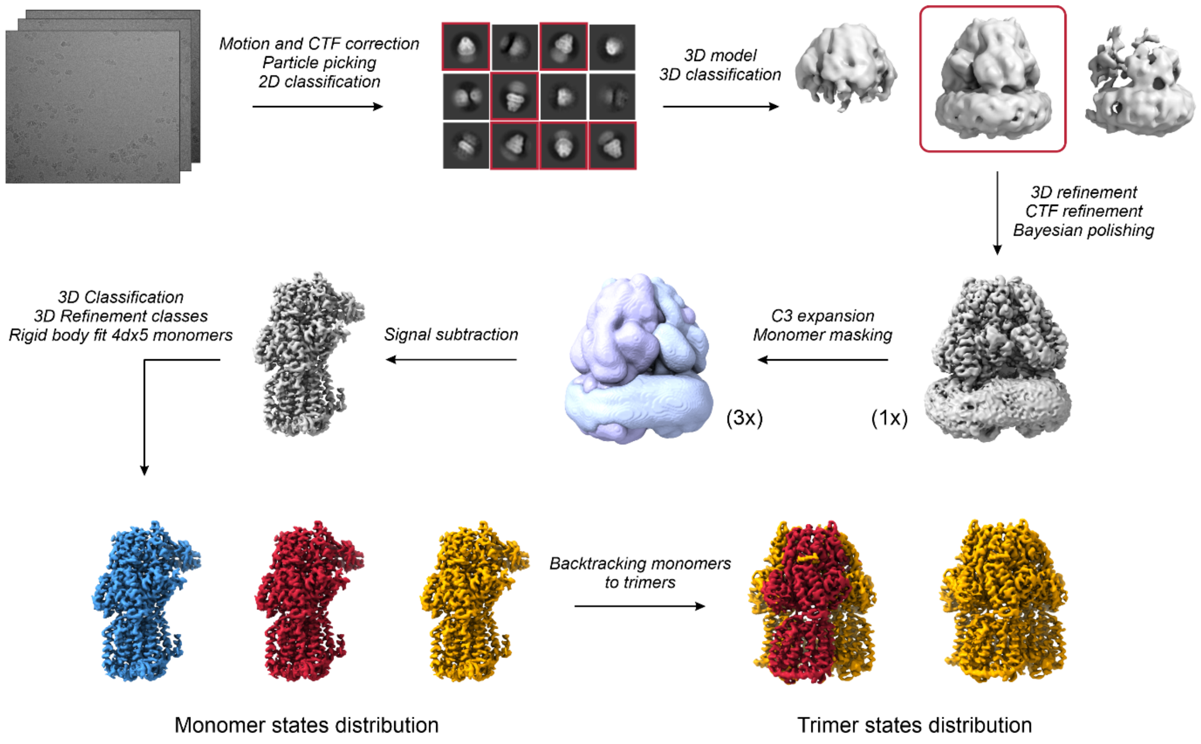


**Supplementary Figure 7: Processing pipeline for cryo-EM datasets.** Micrographs from vitrified samples were recorded on a Titan Krios cryo-TEM operating at 300 kV. Beam-induced motion correction and CTF estimation were performed prior to template based automated particle picking. Several iterative rounds of 2D and 3D classification were performed to remove poor-quality particles, and a 3D volume of the trimer was reconstituted. Utilising the C3 pseudosymmetry through the central axis of the protein, a symmetry expansion was performed, and a soft monomer mask was used to subtract the signal of the two other monomers. Several 3D classification rounds were performed to obtain the maximum number of classes (minimum 3) with the best resolution. Class averages were refined. The conformational state of the refined 3D volumes was assessed by comparison with the AcrB states from the best resolved (1.9 Å) AcrB crystallographic structure (PDB ID: 4dx5). A custom MATLAB script was used to calculate the trimer composition based on the position of the monomer particles.

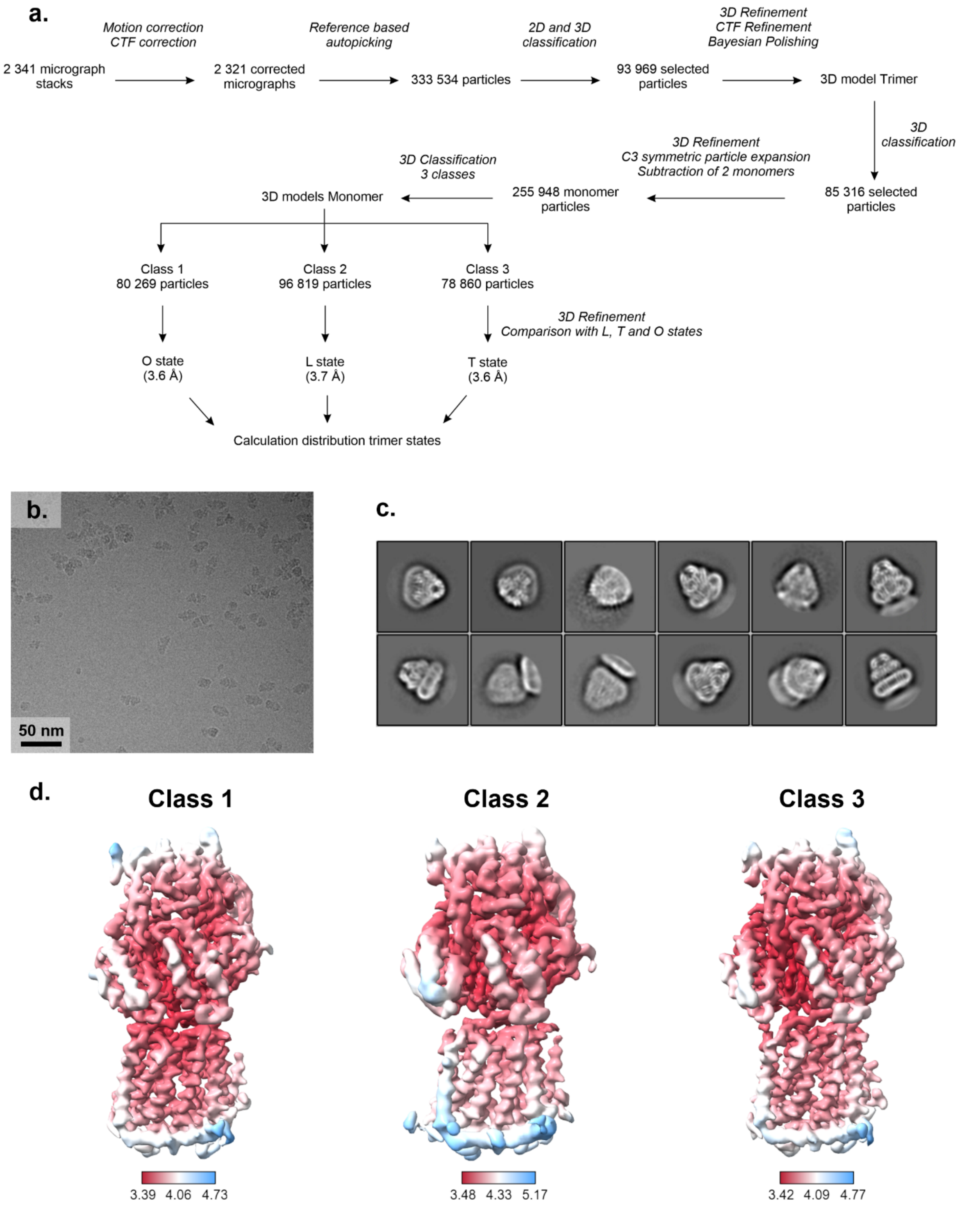


**Supplementary Figure 8: Cryo-EM data of AcrB wildtype solubilised in DDM (a.)** Processing pipeline for evaluation of the distribution of conformational states. The processing was performed in Relion [12]. The conformational state of each class was determined by comparison with the three monomers of the best resolved asymmetric LTO structure of AcrB wildtype (PDB ID: 4dx5). Representative micrograph and 2D classes from the dataset are shown in (**b.**). and (**c.**), respectively. The local resolution of the 3D volumes of the monomer class averages is depicted in (**d.**)

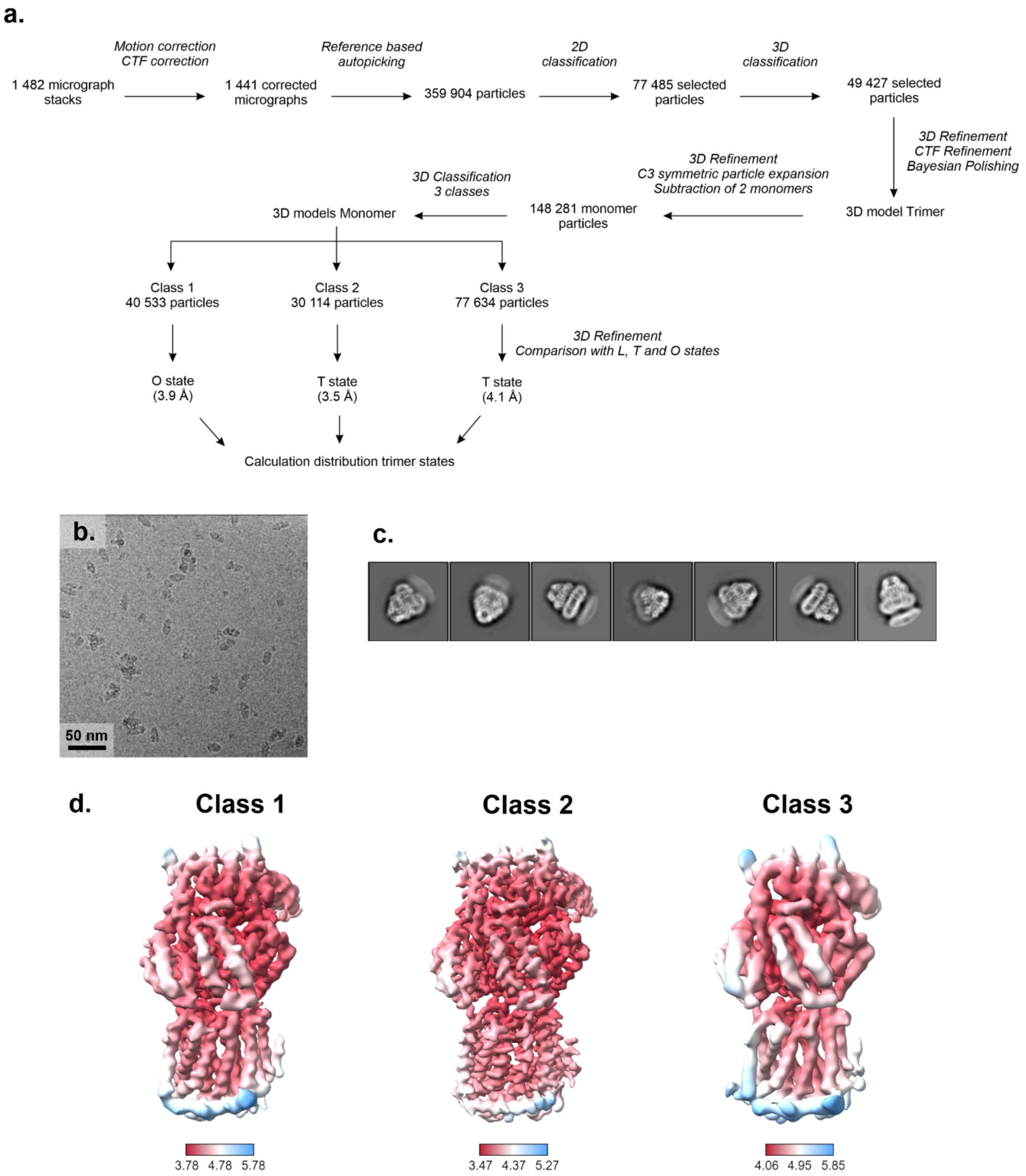


**Supplementary Figure 9: Cryo-EM data of AcrB V612F solubilised in DDM. (a.**) Processing pipeline for evaluation of the distribution of conformational states. The processing was performed in Relion [12]. The conformational state of each class was determined by comparison with the three monomers of the best resolved asymmetric LTO structure of AcrB wildtype (PDB ID: 4dx5). Representative micrograph and 2D classes from the dataset are shown in (**b.**). and (**c.**), respectively. The local resolution of the 3D volumes of the monomer class averages is depicted in (**d.**)

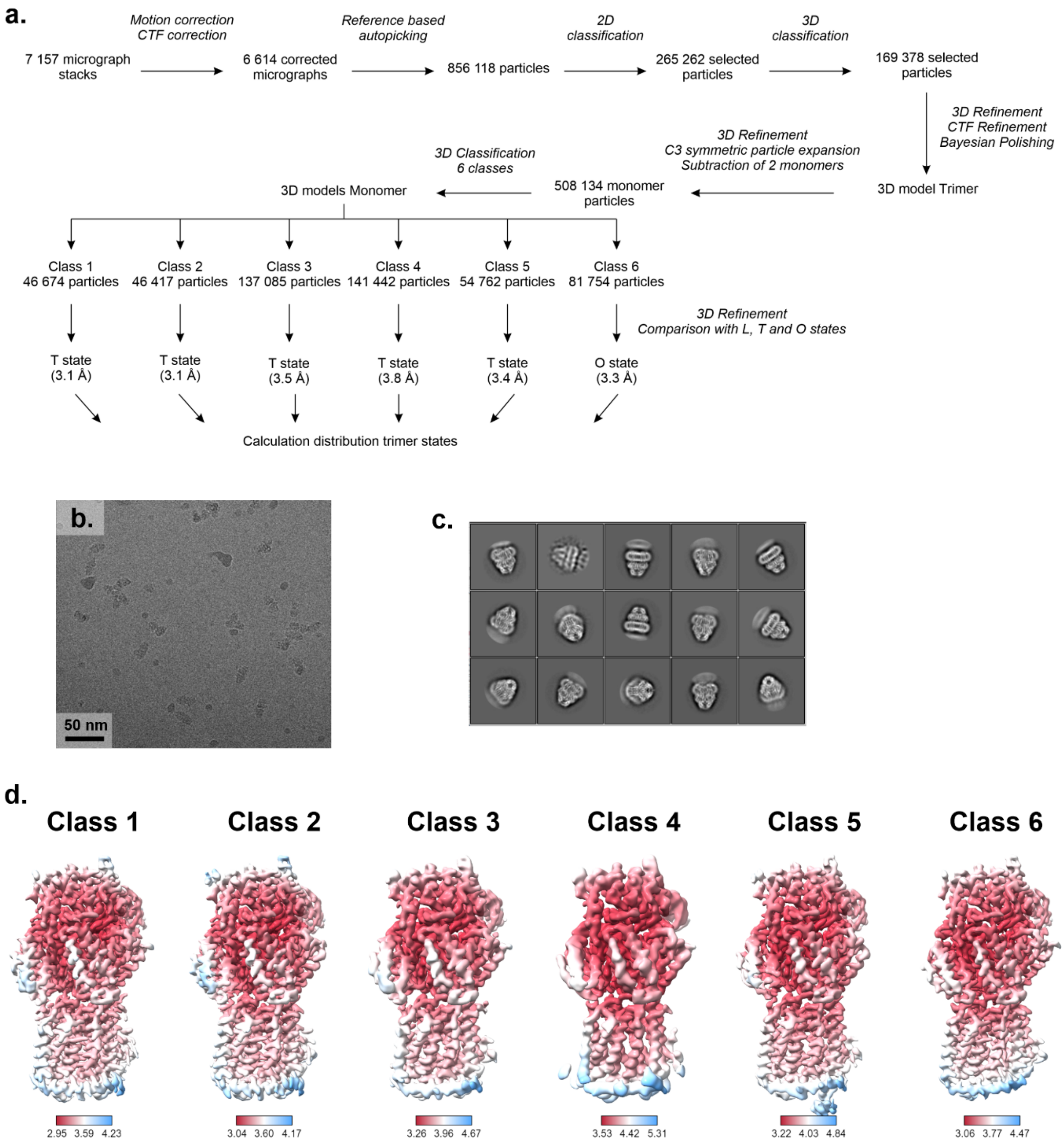


**Supplementary Figure 10: Cryo-EM data of AcrB V612W solubilised in DDM. (a.**) Processing pipeline for evaluation of the distribution of conformational states. The processing was performed in Relion [12]. The conformational state of each class was determined by comparison with the three monomers of the best resolved asymmetric LTO structure of AcrB wildtype (PDB ID: 4dx5). Representative micrograph and 2D classes from the dataset are shown in (**b.**). and (**c.**), respectively. The local resolution of the 3D volumes of the monomer class averages is depicted in (**d.**)

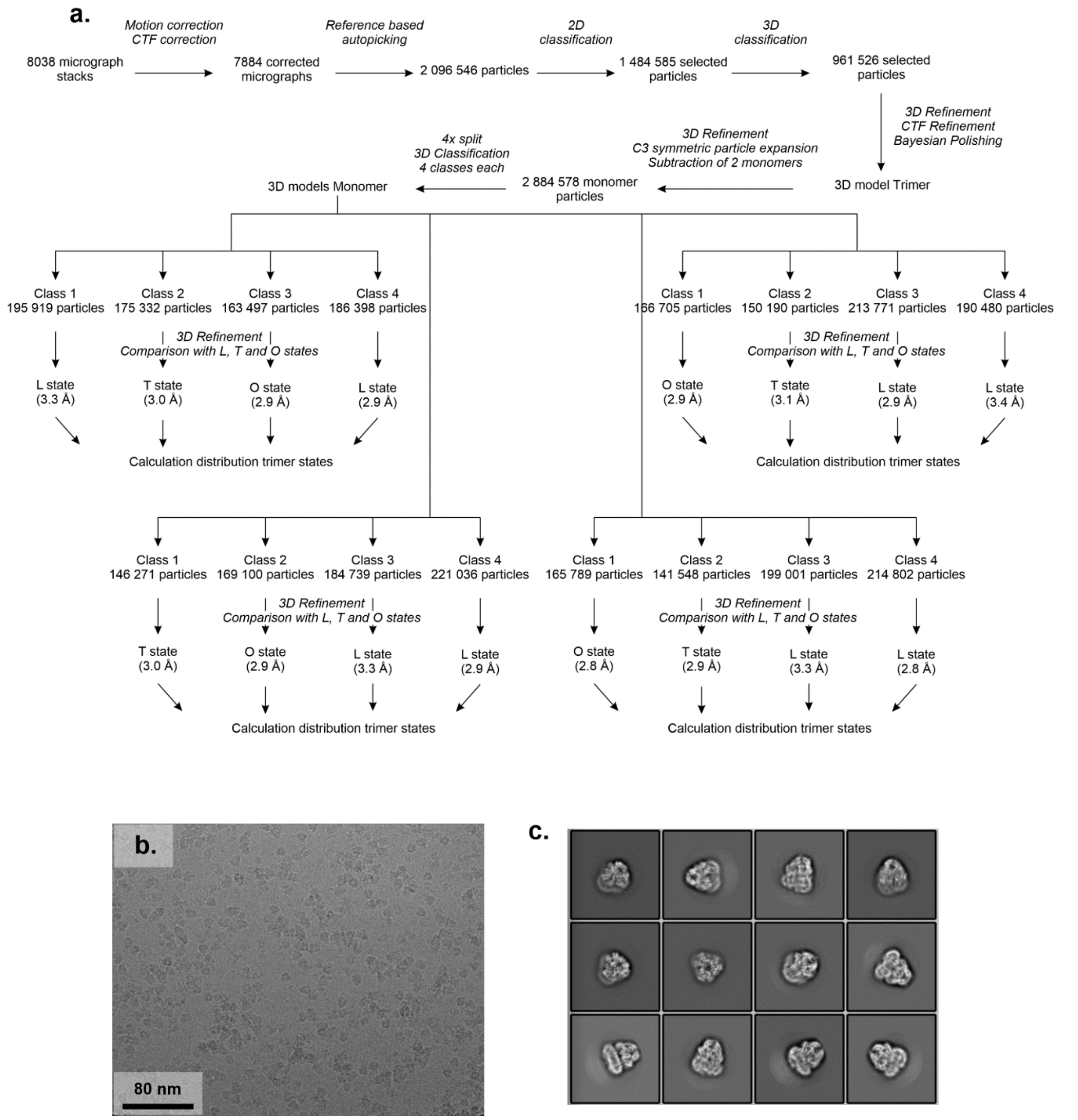


**Supplementary Figure 11: Cryo-EM data of AcrB wildtype reconstituted in salipro nanodiscs. (a.)** Processing pipeline for evaluation of the distribution of conformational states. The processing was performed in Relion [12]. Due to the high number of particles, the dataset was split in four parts, that were processed independently in parallel. The conformational state of each class was determined by comparison with the three monomers of the best resolved asymmetric LTO structure of AcrB wildtype (PDB ID: 4dx5). Representative micrograph and 2D classes from the dataset are shown in (**b.**). and (**c.**), respectively. The local resolution of the 3D volumes of the monomer class averages is depicted in Supplementary Figure 12.

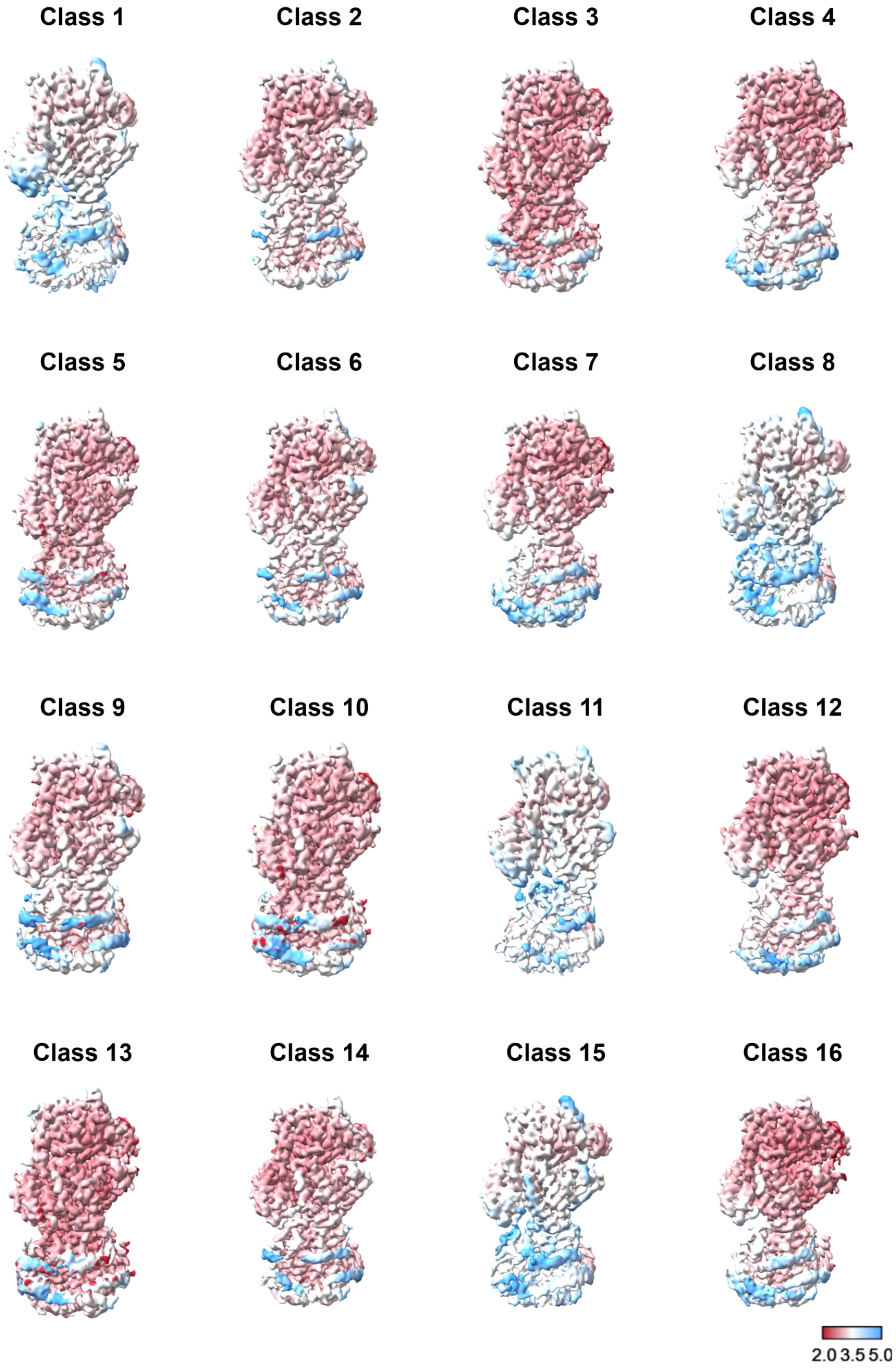


**Supplementary Figure 12: Local resolution of the monomer classes from the AcrB wildtype Salipro nanodiscs sample.** The monomer volumes were determined as described in Supplementary Figure 11.

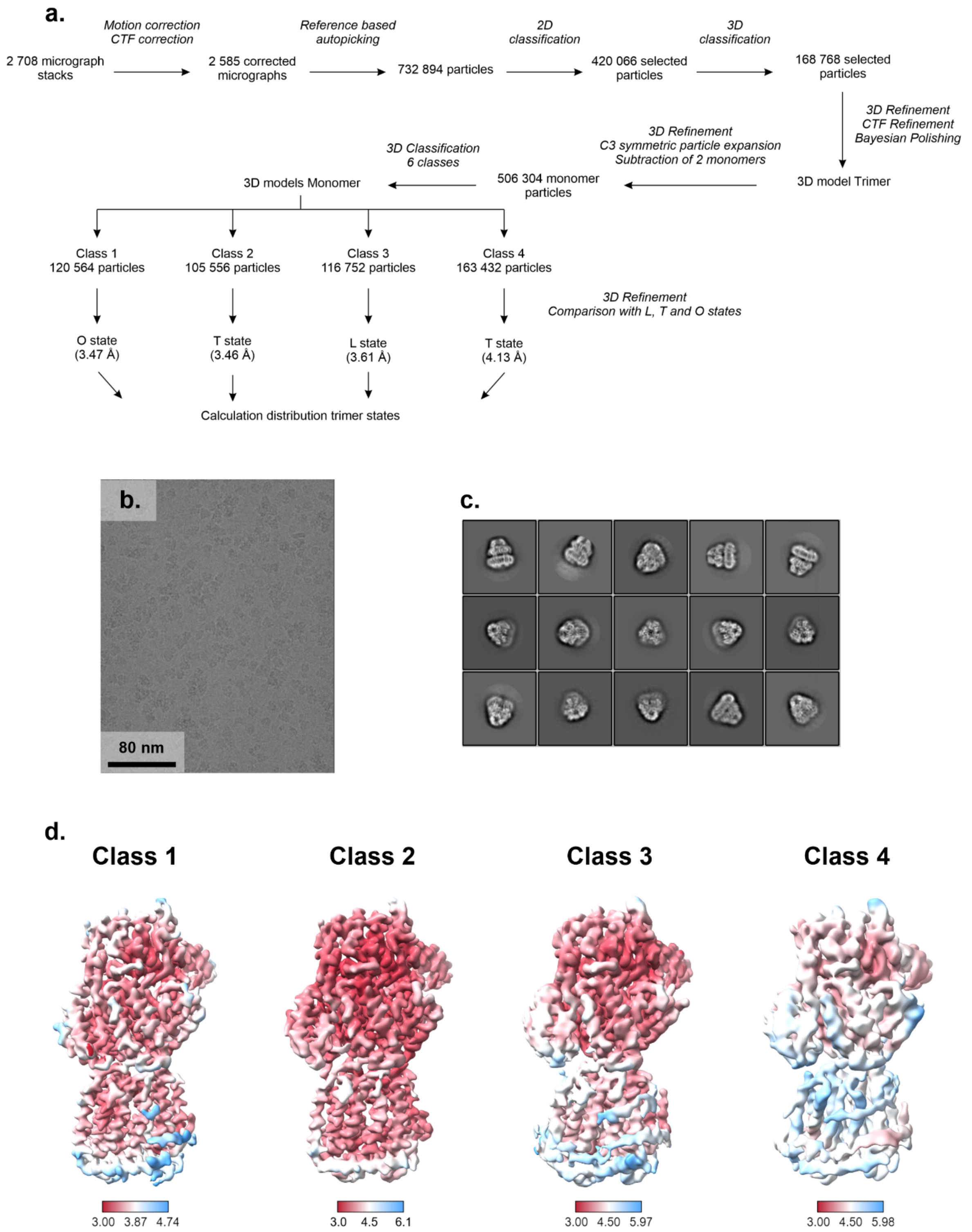


**Supplementary Figure 13: Cryo-EM data of AcrB V612F reconstituted in salipro nanodiscs. (a.)** Processing pipeline for evaluation of the distribution of conformational states. The processing was performed in Relion [12]. The conformational state of each class was determined by comparison with the three monomers of the best resolved asymmetric LTO structure of AcrB wildtype (PDB ID: 4dx5). Representative micrograph and 2D classes from the dataset are shown in (**b.**). and (**c.**), respectively. The local resolution of the 3D volumes of the monomer class averages is depicted in (**d.**)

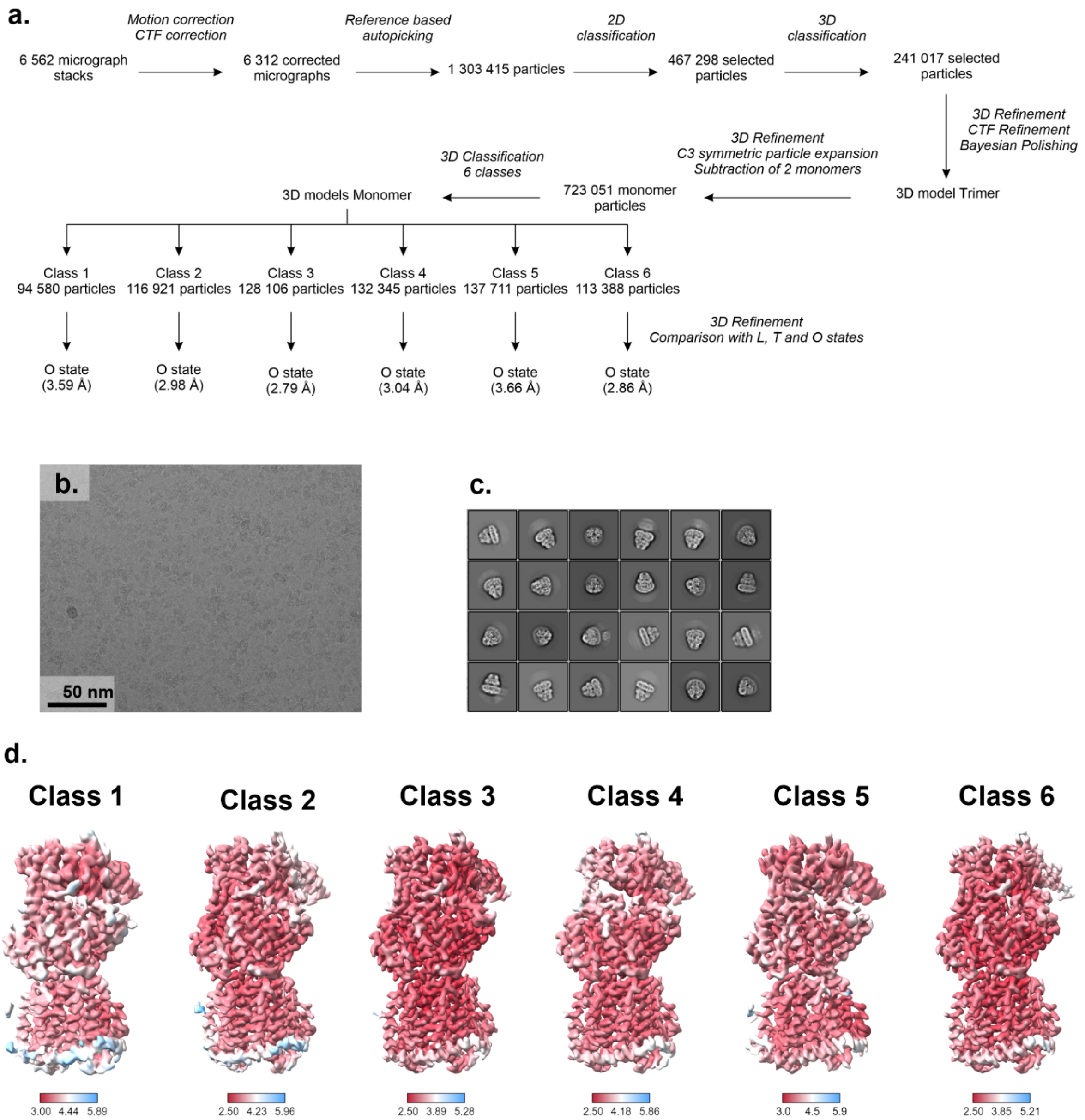


**Supplementary Figure 14: Cryo-EM data of OqxB wildtype reconstituted in salipro nanodiscs. (a.)** Processing pipeline for evaluation of the distribution of conformational states. The processing was performed in Relion [12]. The conformational state of each class was determined by comparison with the three monomers of the best resolved asymmetric LTO structure of AcrB wildtype (PDB ID: 4dx5). Representative micrograph and 2D classes from the dataset are shown in (**b.**). and (**c.**), respectively. The local resolution of the 3D volumes of the monomer class averages is depicted in (**d.**)

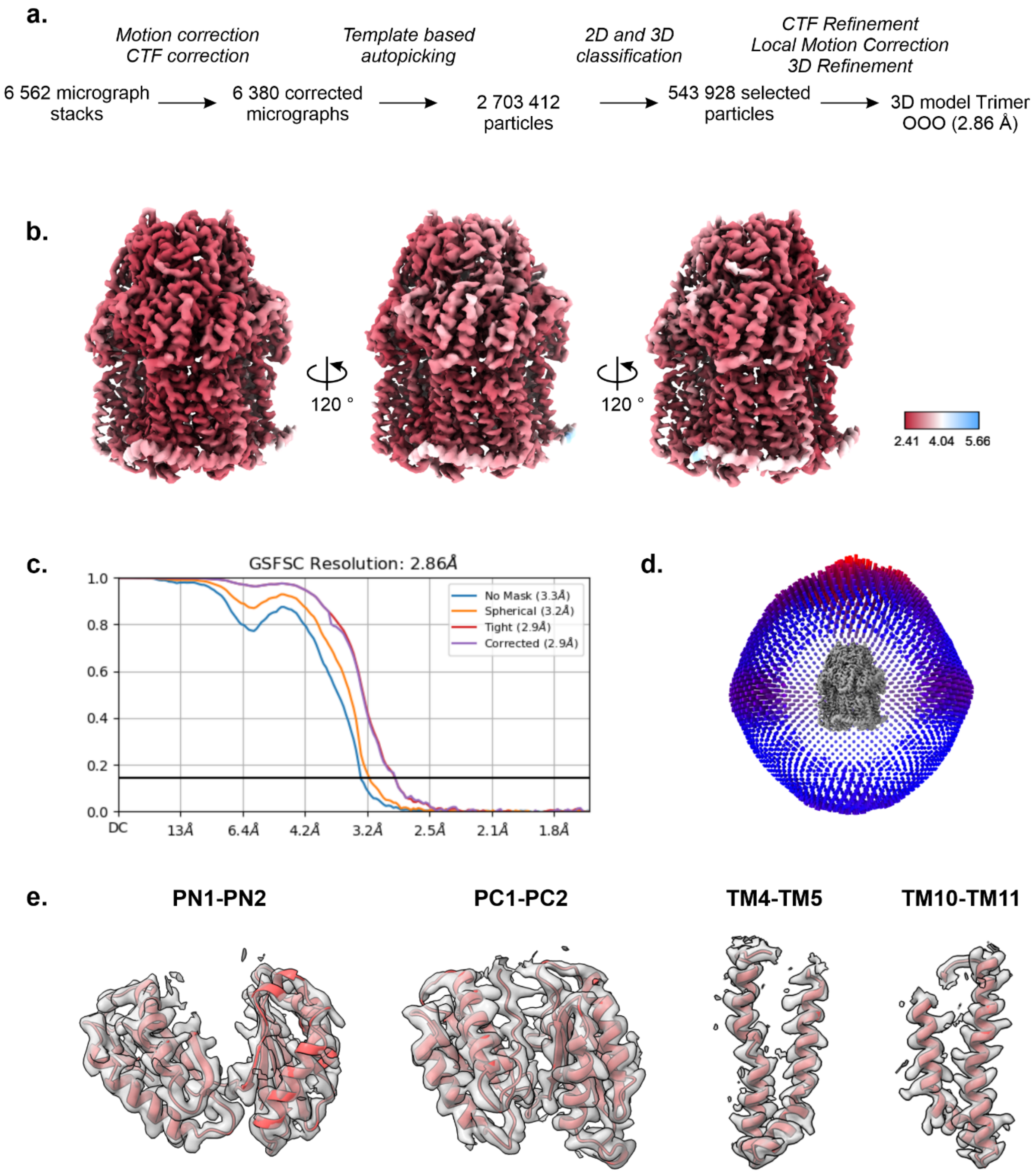


**Supplementary Figure 15: Processing pipeline for structure determination of the OqxB trimer.** The same dataset as in Supplementary Figure 14 was processed again in Cryosparc [13]. The template-based picking resulted in more good particles compared to the processing in Relion [3] leading to a better resolution in the final 3D trimer map, therefore this model was used for structure building. The processing pipeline is depicted in (**a**). The model was processed without imposed symmetry. The local resolution of the trimer, gold standard FSC curves and the angular distribution of the particles are shown in (**b.**), (**c.**) and (**d.**) respectively. Representative key regions in the porter domain (PN1, PN2, PC1 and PC2 subdomains) and the transmembrane domain (helices TM4, TM5, TM10 and TM11) are displayed as an overlay of the map (semi-transparent grey) and the model (red) in (**e.**).

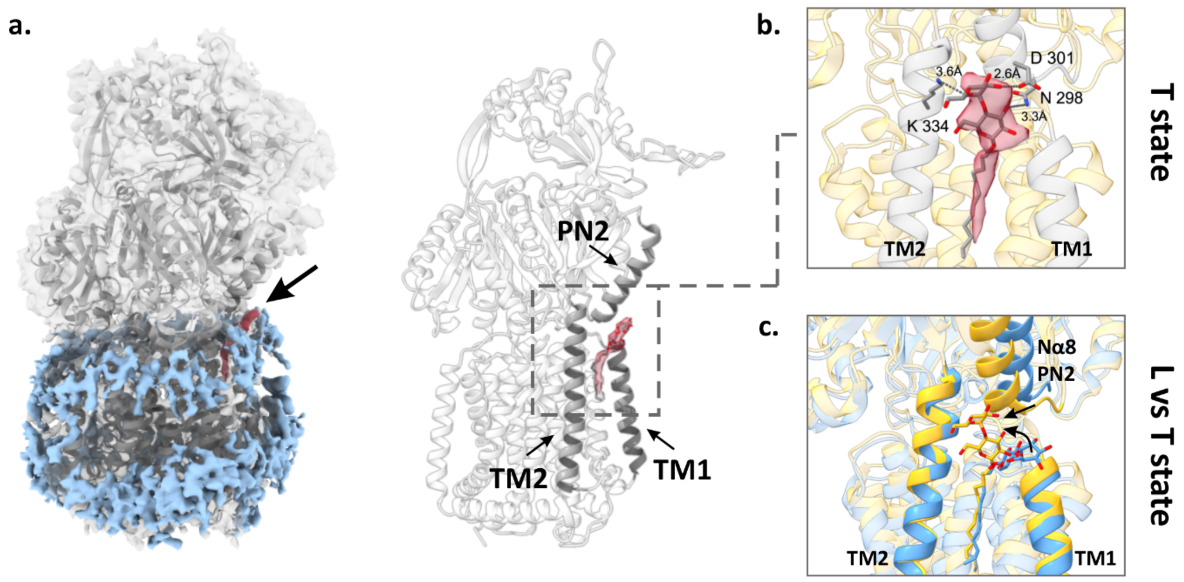


**Supplementary Figure 16: DDM binding in the transmembrane domain of AcrB.** In several of the monomer classes in the cryo-EM datasets of detergent solubilised AcrB a DDM density was observed in the groove between transmembrane helices TM1 and TM2. This density is shown exemplary for class 1 of the V612W dataset. (**a**) The 3D electron density map of V612W, class 1 (left panel) was overlayed with the structure model of the AcrB T monomer with bound DDM (PDB ID: 6zoe). The micelle densities surrounding the TMD are shown in blue. The DDM molecule in the TM1/TM2 groove is depicted in red and indicated by the arrow. TM1, TM2 and the Nα8 helix from the PN2 subdomain, that coordinate the DDM molecule (red), are highlighted in the right panel. A closer zoom of the detergent binding site is depicted in (**b**). The structure model of AcrB in the T state (PDB ID: 6zoe) fitted in the density map is shown in yellow with the TM1, TM2 and Nα8 helices highlighted in grey. DDM and the residues N298, D301 and K334 that coordinate the maltose rings are depicted as sticks coloured by element with carbon atoms in grey, oxygen in red and nitrogen in blue. The DDM density from the cryo-EM map is displayed in red. (**c**) Comparison of the DDM orientation in the TM1/TM2 groove of the L and T states. The figure shows an overlay of the L (blue) and T (yellow) states of DDM bound crystallographic AcrB structures (PDB ID: 4dx5 and 6zoe). The TMD helices TM1 and TM2 and the Nα8 helix of the PN2 subdomain are indicated. DDM is shown as sticks coloured by atom type with carbon atoms in blue (L state) or yellow (T state) and oxygen atoms in red. The shifts of the maltose ring of DDM and the PN2 subdomain from the L to the T state are indicated by the arrows.

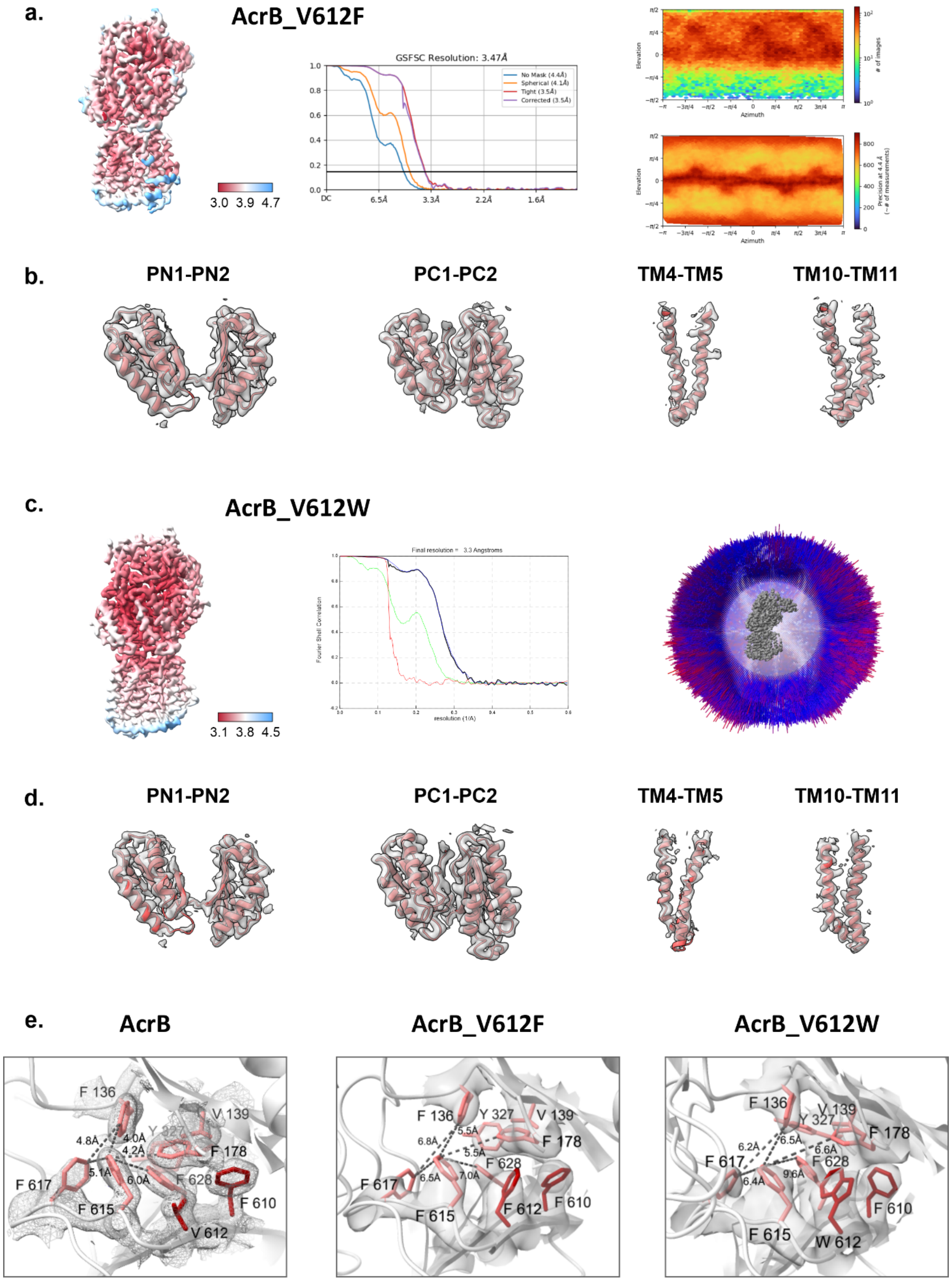


**Supplementary Figure 17: Deep binding pocket of AcrB wildtype, V612F and V612W in the O state.** Based on the best resolved O state monomer maps, structural models were built for the V612F and V612W variants of AcrB in the O state. The structural model of AcrB_V612F was based on the class 1 monomer from the dataset in SP-ND. The processing pipeline is depicted in detail in Supplementary Figure 13. The local resolution of the monomer, gold standard FSC curves and the angular distribution of the particles are shown in (**a**). Representative

key regions in the porter domain (PN1, PN2, PC1 and PC2 subdomains) and the transmembrane domain (helices TM4, TM5, TM10 and TM11) are displayed as an overlay of the map (semi-transparent grey) and the model (red) in (**b**). The structural model of AcrB_V612W was based on the class 6 monomer from the dataset in DDM. The processing pipeline is depicted in detail in Supplementary Figure 10. The local resolution of the monomer, gold standard FSC curves and the angular distribution of the particles are shown in (**c**). Representative key regions in the porter domain (PN1, PN2, PC1 and PC2 subdomains) and the transmembrane domain (helices TM4, TM5, TM10 and TM11) are displayed as an overlay of the map (semi-transparent grey) and the model (red) in (**d**). The conserved hydrophobic pocket residues in the DBP of AcrB wildtype (AcrB, PDB ID: 4dx5) and in the structures of the O state monomers of AcrB_V612F and AcrB_V612W are compared in (**e**). The residues at positions 610 and 612 are highlighted in darker red. The dashed lines indicate the distances measured between selected DBP residues. Crystallographic $2F_o$-$F_c$ density (AcrB) depicted as a mesh at 1σ. Cryo-EM maps are depicted as solid surface with contour level 0.0828 (AcrB_V612F) and 0.0096 (AcrB_V612W).

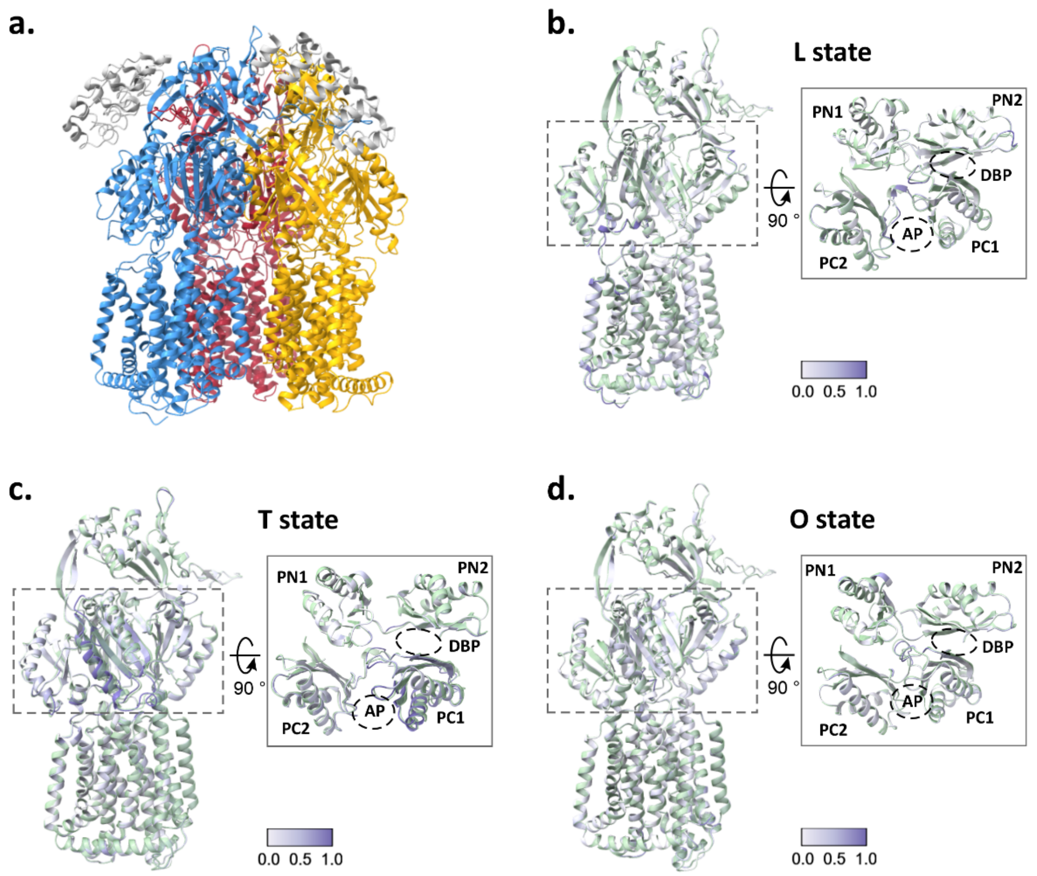


**Supplementary Figure 18: Structure of AcrB V612N in the LTO state.** (**a.**) Structure of the AcrB V612N variant solved using electron density maps derived from X-ray data of crystals grown in the $P2_12_12_1$ space group. The structure was solved as an asymmetric AcrB trimer with two bound DARPins. Comparison with the asymmetric structure of AcrB wildtype (PDB ID: 4dx5) allowed to identify the V612N trimeric state as LTO. Colours: blue – L state, yellow – T state, red – O state, grey – DARPin. (**b.-d.**) Superimposition of each of the V612N monomers with the best fitting AcrB wildtype L, T, or O states is shown as side views. The V612N monomer is coloured by RMSD to the wildtype as indicated in the respective colour key, the wildtype monomer (derived from PDB ID: 4dx5) is coloured in green. Insets: top view on the AcrB porter domain with indication of the PN1, PN2, PC1, and PC2 subdomains. Dashed ovals indicate the location of the access pocket (AP) and deep binding pocket (DBP).

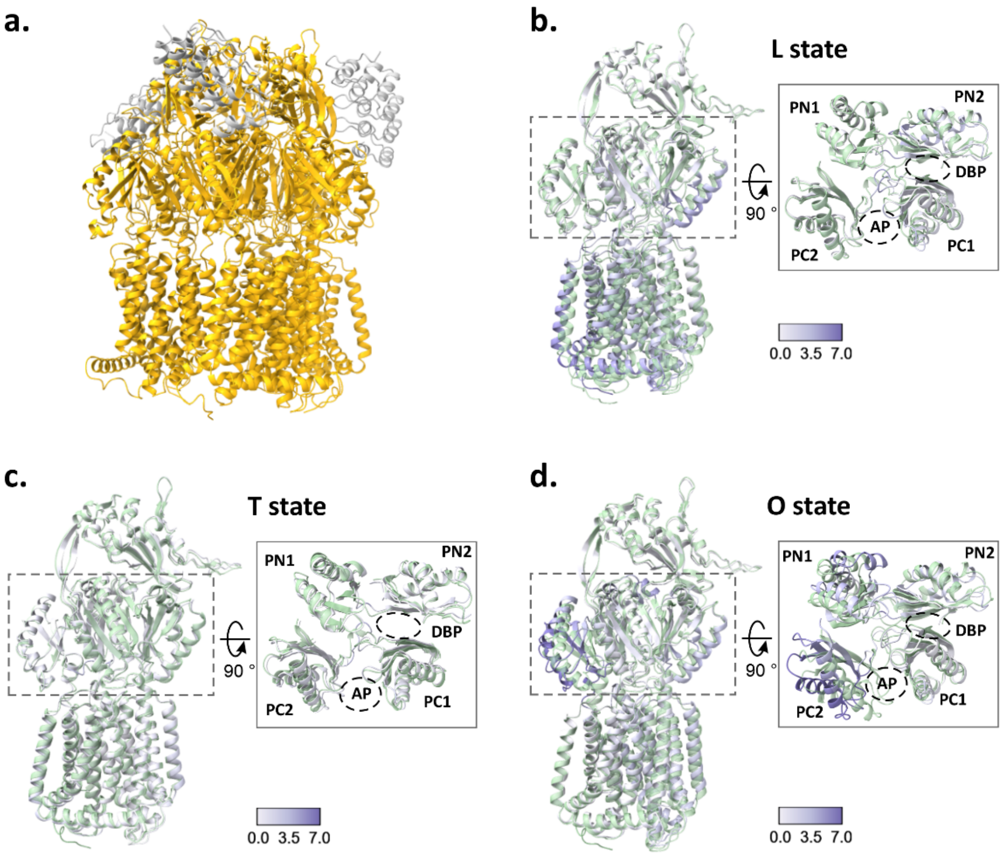


**Supplementary Figure 19: Structure of AcrB V612N in the TTT state.** (**a.**) Structure of the AcrB V612N variant solved using electron density maps derived from X-ray data of crystals grown in the I23 space group. The structure was solved as a symmetric AcrB trimer with three identical chains. One DARPin molecule is associated with each chain. Comparison with the asymmetric structure of AcrB wildtype (PDB ID: 4dx5) allowed to identify the V612N trimeric state as TTT. Colours: yellow – AcrB, grey – DARPin. (**b.-d.**) Superimposition of one representative V612N monomer with each of the monomer states of the AcrB wildtype (L, T, or O) is shown as side views. The V612N monomer is coloured by RMSD to the wildtype as indicated in the respective colour key, the wildtype monomer (derived from PDB ID: 4dx5) is coloured in green. Insets: top view on the AcrB porter domain with indication of the PN1, PN2, PC1, and PC2 subdomains. Dashed ovals indicate the location of the access pocket (AP) and deep binding pocket (DBP).

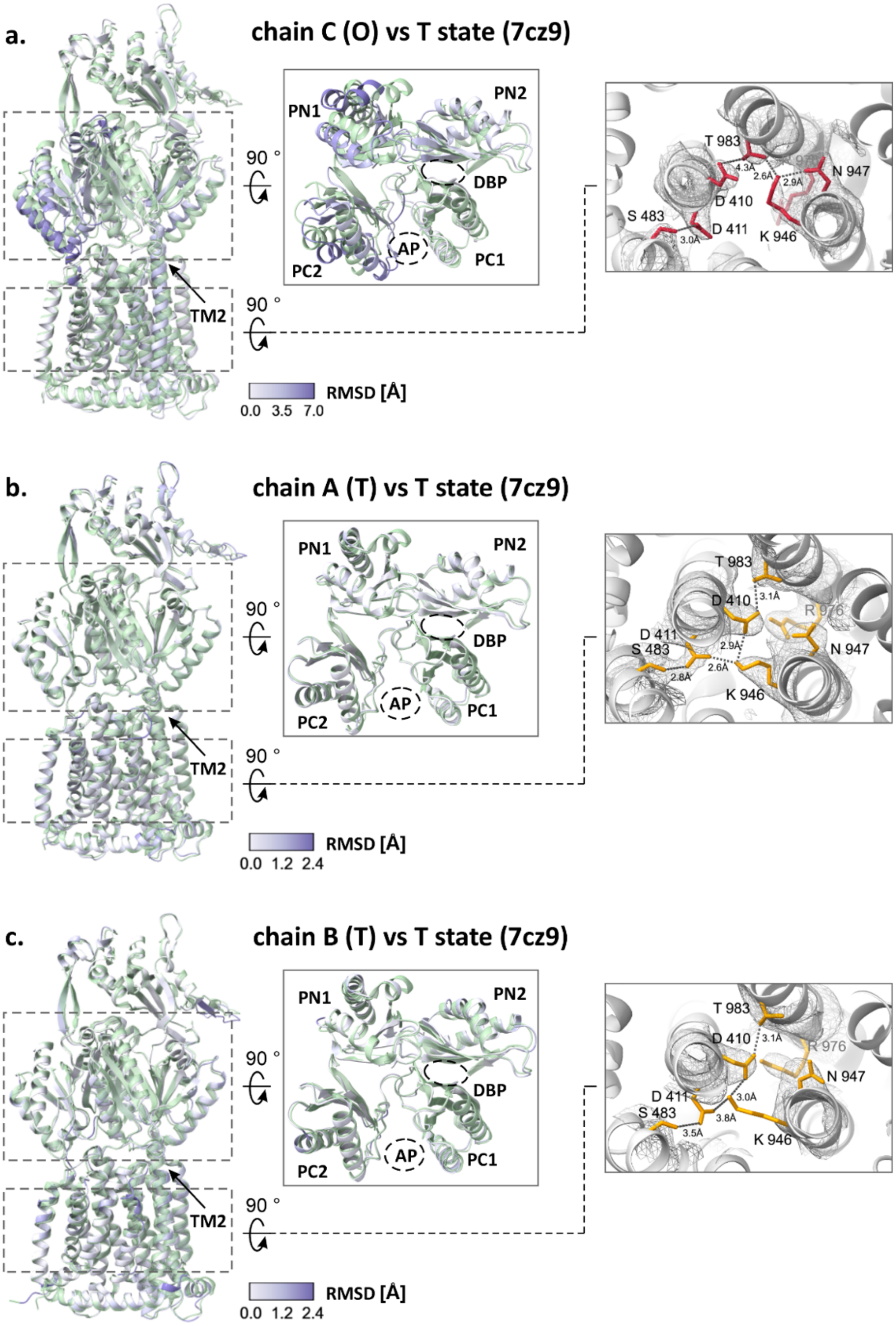


**Supplementary Figure 20: Crystallographic structure of OqxB in the TTO state.** Each chain (**a-c**) of the crystallographic structure of OqxB presented in this study was overlayed with the T state of OqxB from the previously published TTT structure (PDB ID: 7cz9). Left panel: Overlay of the full-length monomers. Middle panel: a top view of the PD. The PD subdomains and the TM2 are indicated. The TTO structure is coloured by the RMSD to the T state according to the respective colour key. OqxB in the T state (PDB ID: 7cz9) is coloured green. Right panel: Proton translocation network in the TTO structure. The central titratable residues of the proton translocation network are shown as sticks in red (O state) and yellow (T states) for the TTO structure. Crystallographic $2F_o$-$F_c$ densities are shown as a mesh at σ 1. The distances between the residues are indicated by the dashed lines.

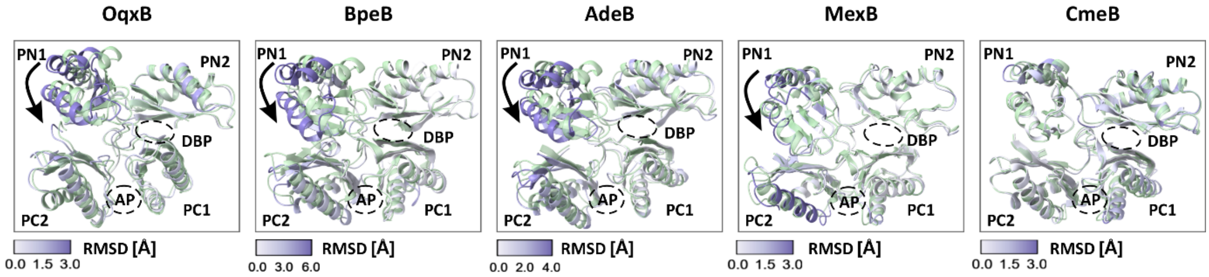


**Supplementary Figure 21: Comparison of the O and O* states across RND multidrug efflux pumps.** The monomers in the O and the O* states of OqxB (this study), BpeB (PDB ID: 7wls), AdeB (PDB ID: 7kgh), MexB (PDB ID: 6ta6 and 6t7s) and CmeB (PDB ID: 5lq3) were overlayed. The O* state is coloured green, and the O state is coloured by the RMSD to O* as indicated in the respective colour key. The figure shows a top view of the porter domain. The PC1, PC2, PN1 and PN2 subdomains and the substrate binding pockets (AP, DBP) are labelled. The shift of the PN1 subdomain in the O* state is indicated by the arrows.

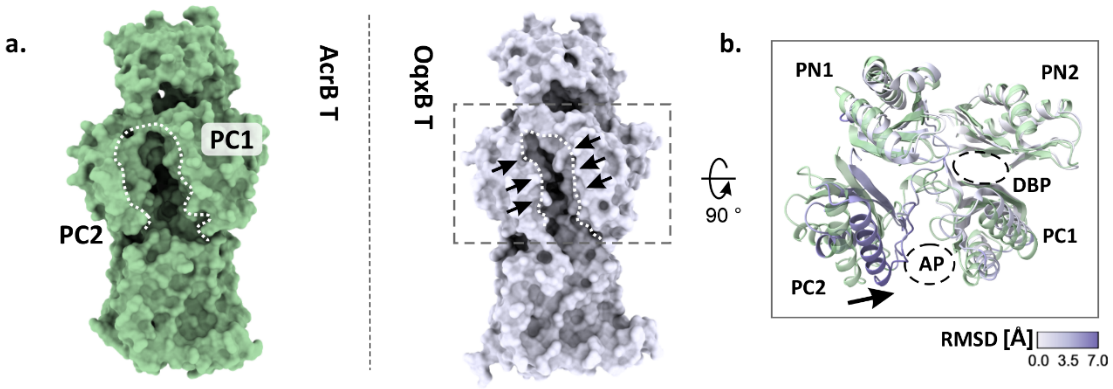


**Supplementary Figure 22: Comparison of the T state of OqxB and AcrB.** (**a**) Surface representation of AcrB (PDB ID: 4dx5, left panel, green) and OqxB (PDB ID 7cz9, right panel, grey) in the T state. The entrance of the AP, flanked by the helices of the PC1 and PC2 subdomains, is indicated. Arrows indicate the reduced cleft in OqxB. The T states of AcrB and OqxB were aligned, and a top view of the porter domains is shown in (**b**). The subdomains (PN1, PN2, PC1, PC2) and the substrate binding pockets (AP, DBP) are indicated. AcrB is coloured green and OqxB is coloured by the RMSD to AcrB according to the displayed colour key. The shift of PC2 observed in OqxB is indicated by the arrow.

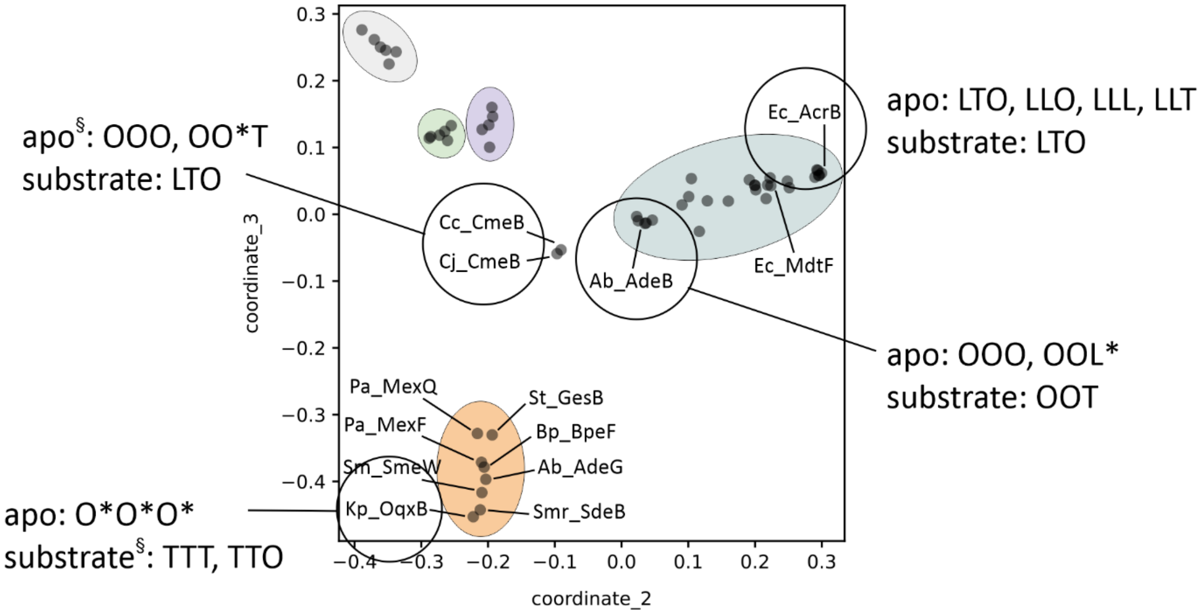


**Supplementary Figure 23: Distribution of conformational states across HAE-1 RND efflux pumps.** The conformational states observed for apo and substrate bound *E. coli* AcrB (this study), *A. baumannii* AdeB [1,14], *Campylobacter* CmeB [15,16] and *K. pneumoniae* OqxB ([5] and this study) are summarised in the PaSi map from Fig. 1. The cryo-EM analysis of AcrB suggests an intrinsically flexible trimer that exists in an equilibrium between the LTO, LLL, LLT and LLO states. In contrast, AdeB that marks the far end of the AcrB cluster shows a reduced heterogeneity with only the OOO and OOL* conformations in the apo state. The L* state resembles the T state, but in contrast to T the DBP is closed. Similarly to AdeB, for apo CmeB, OOO and OO*T conformations have been described. Finally, for OqxB, a homogenous conformation with a single closed state (O*O*O*) was found for the apo protein. Differences also seem to be present in the conformations adopted in the presence of substrates. For AcrB in presence of DDM, the LTO state was predominant in the sample, whereas all further possible trimer compositions were found at low frequencies (less than 10 %). AdeB in the presence of ethidium adopted the OOT state. The LTO and OO*T states were also found in around 5% of the particles. CmeB adopted the LTO state in the presence of several different drugs. OqxB adopts the TTT and TTO states in the presence of DDM and binding of the detergent in the DBP was observed. § indicates crystallographic structures. Structural information for all other states is derived from cryo-EM studies. For the cryo-EM data, the conformations adopted by the major fraction of the particles (> 10 %) is displayed.

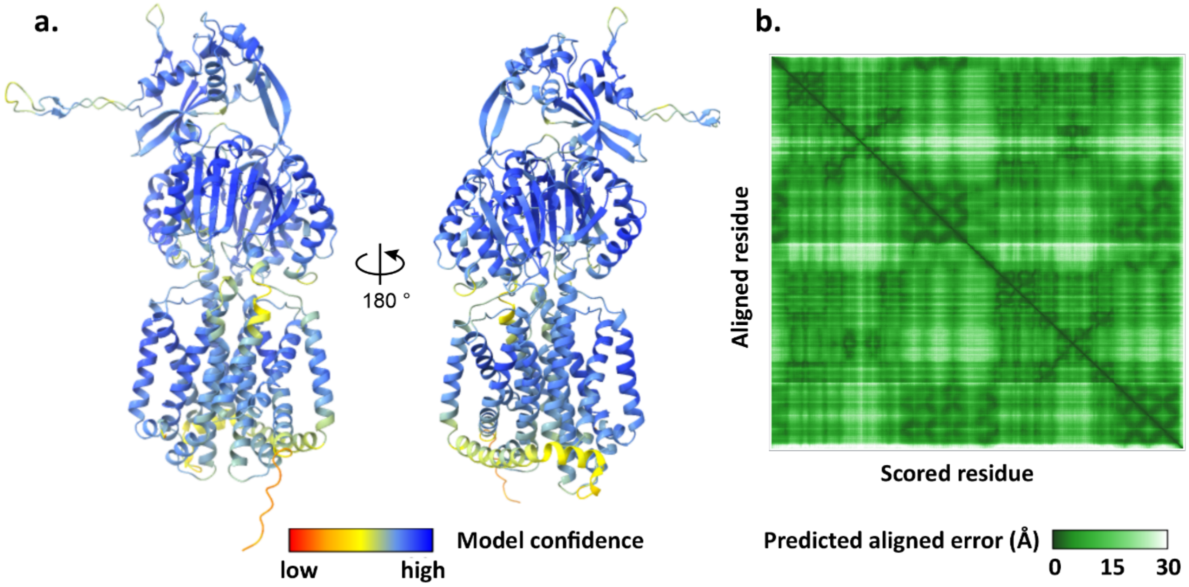


**Supplementary Figure 24: AlphaFold structure model of OqxB.** The model is based on the AlphaFold structure available in the AlphaFold Structure Database (https://alphafold.ebi.ac.uk) under UniProt accession number U5U6L7 (accession date: January 8th, 2024). The model (AF-U5U6L7-F1-v4) has a sequence length of 1052 and an average pLDDT of 88.69 (high).

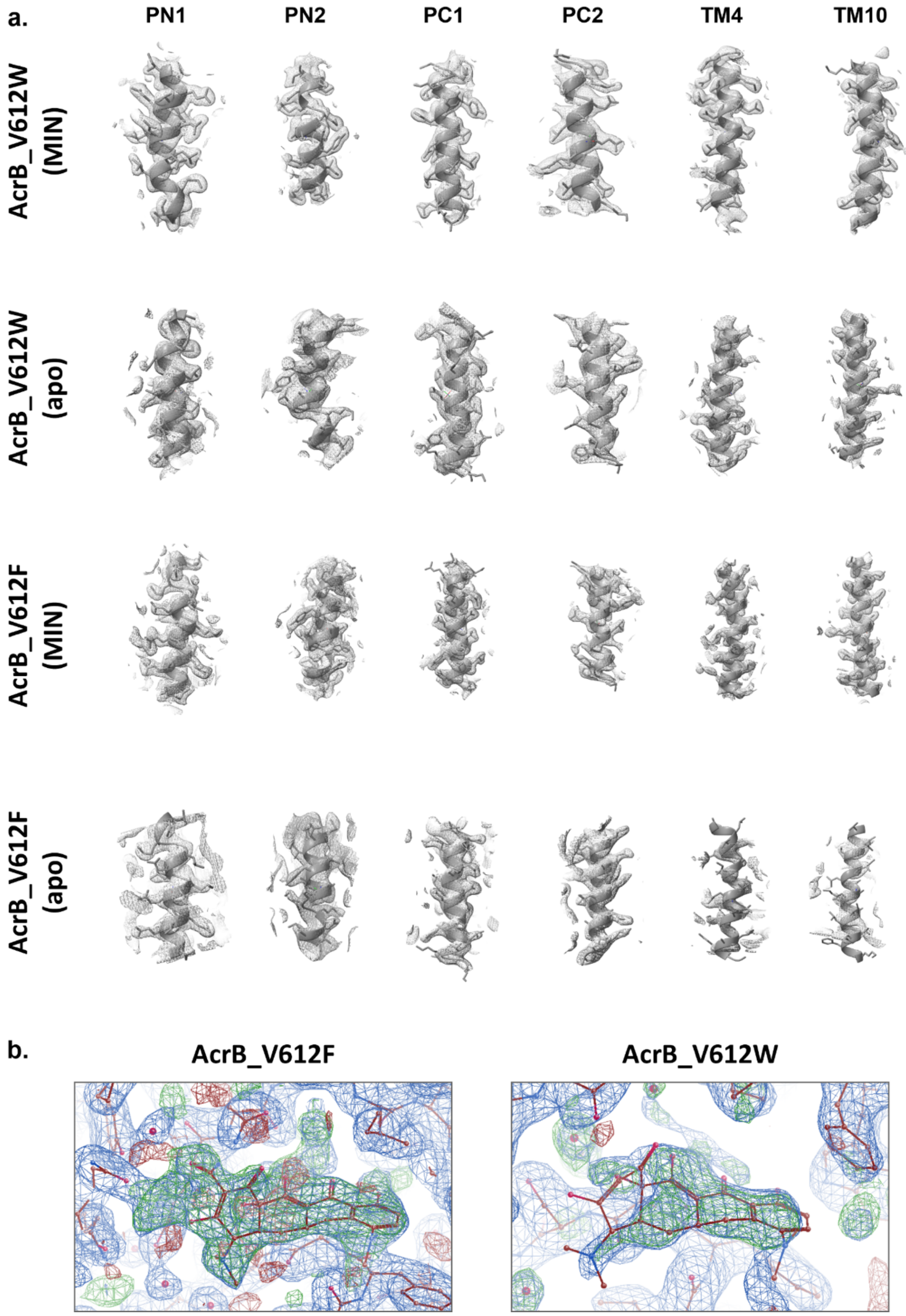


**Supplementary Figure 25: Representative regions of the crystallographic structures of AcrB_V612F and V612W. (a.)** Representative key regions in the porter domain (PN1, PN2, PC1 and PC2 subdomains) and the transmembrane domain (helices TM4, TM10) are displayed as an overlay of the crystallographic $2F_o$-$F_c$ maps (grey mesh displayed at $1\sigma$) and the structural model (grey cartoon). (**b.**) Ligand densities for minocycline in the crystallographic AcrB_V612F and V612W structures. Crystallographic $F_o$-$F_c$ density maps (positive values: green, negative values: red, displayed at $3\sigma$) are overlayed with $2F_o$-$F_c$ maps (blue, displayed at $1\sigma$) after modelling of the proteinogenic densities. The structural model is displayed as sticks.

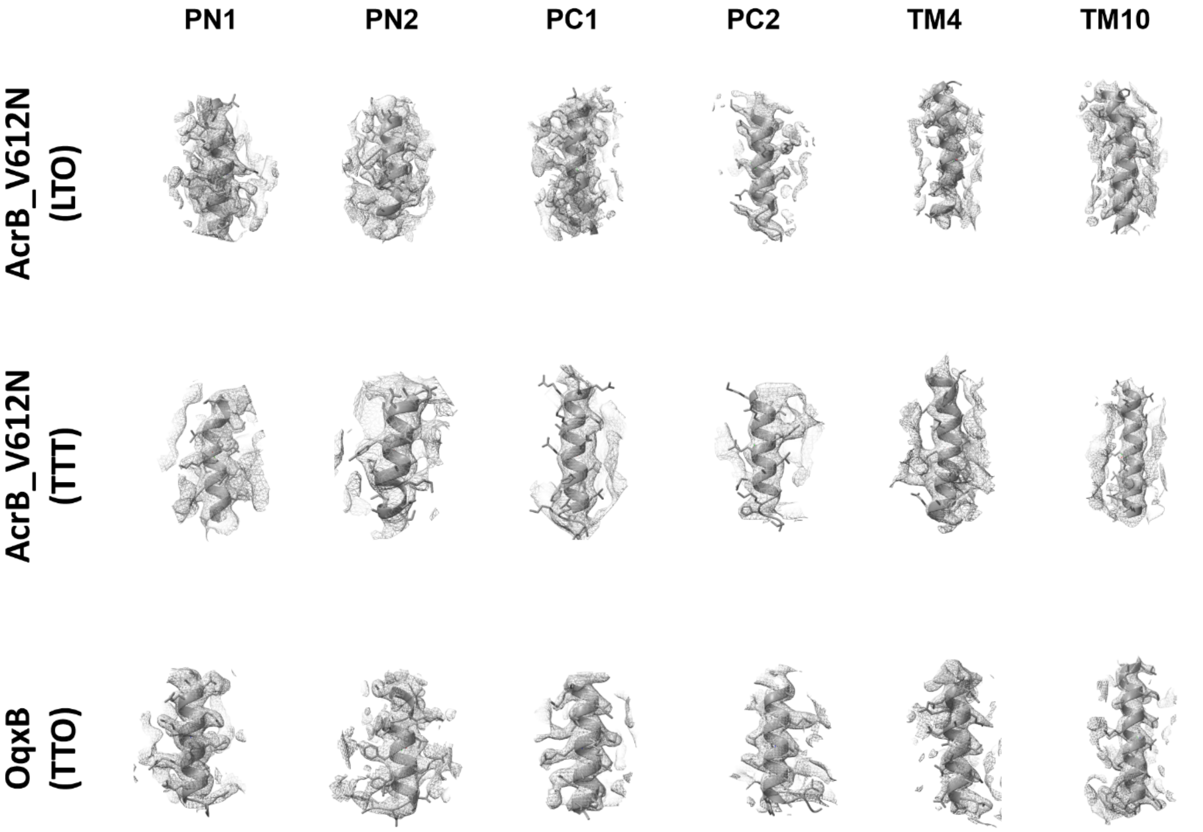


**Supplementary Figure 26: Representative regions of the crystallographic structures of AcrB_V612N and OqxB in the TTO state.** Representative key regions in the porter domain (PN1, PN2, PC1 and PC2 subdomains) and the transmembrane domain (helices TM4, TM10) are displayed as an overlay of the crystallographic $2F_o$-$F_c$ maps (grey mesh displayed at 1$\sigma$) and the structural model (grey cartoon).

**Supplementary Tables**

# Conformational plasticity across phylogenetic clusters of RND multidrug efflux pumps and its impact on substrate specificity

Mariya Lazarova[1], Thomas Eicher[1], Clara Börnsen[2], Hui Zeng[1], Mohd Athar[3], Ui Okada[4], Eiki Yamashita[5], Inga M. Spannaus[1], Max Borgosch[1], Hi-jea Cha[1], Attilio V. Vargiu[3], Satoshi Murakami[4*], Kay Diederichs[6*], Achilleas S. Frangakis[2*], Klaas M. Pos [1*]

[1] Institute of Biochemistry, Goethe-University Frankfurt, Germany

[2] Buchmann Institute for Molecular Life Sciences and Institute of Biophysics, Goethe-University Frankfurt, Germany

[3] Department of Physics, University of Cagliari, Italy

[4] Department of Life Science and Technology, Tokyo Institute of Technology, Yokohama, Japan

[5] Institute for Protein Research, Osaka University, Japan

[6] Department of Biology, University of Konstanz, Germany

* *Corresponding authors*

Klaas M. Pos, pos@em.uni-frankfurt.de

Achilleas S. Frangakis, achilleas.frangakis@biophysik.org

Kay Diederichs, kay.diederichs@uni-konstanz.de

Satoshi Murakami, murakami@bio.titech.ac.jp

**Supplementary Table 1: Uniprot accession numbers of the sequences used for the sequence similarity analysis.** The representative proteins of the HAE-1 RND transporter family in the transporter classification database [17] (accessed 18.08.2023) with addition of the BpeF and CmeB sequences were analysed. The protein sequences deposited in Uniprot (https://www.uniprot.org/, accessed: 18.08.2023) were used. Abbreviations: *Ec: Escherichia coli; Rr: Rhizobium radiobacter; Ab: Acinetobacter baumannii; Ng: Neisseria gonorrhoeae; Pa: Pseudomonas aeruginosa; Pp: Pseudomonas putida; Sm: Stenotrophomonas maltophilia; Ka: Klebsiella aerogenes; Bg: Burkholderia glumae; Cj: Campylobacter jejuni; Cc: Campylobacter coli; Bp: Burkholderia pseudomallei; St: Salmonella typhimurium; Vp: Vibrio parahaemolyticus; Ft: Francisella tularensis; Vc: Vibrio cholerae; Smr: Serratia marcescens; Rs: Ralstonia solanacearum; Abr: Alcanivorax borkumensis; Kp: Klebsiella pneumoniae; Ea: Erwinia amylovora; Hi: Haemophilus influenzae; Pf: Pseudomonas fluorescens*

| Protein | Uniprot ID | Protein | Uniprot ID | Protein | Uniprot ID |
|---|---|---|---|---|---|
| Ec_AcrB | P31224 | Cc_CmeB | A0A1L2IWC1 | Ab_AdeG | Q2FD81 |
| Ec_AcrF | P24181 | Bp_BpeB | Q6VV68 | Rs_AcrB | Q8Y3H0 |
| Rr_IfeB | O68441 | Bp_AmrB | O87936 | Abr_AcrB | Q0VQY6 |
| Ab_AdeE | Q8GKU1 | St_GesB | Q8ZRG9 | Kp_OqxB | C5IZH1 |
| Ng_MtrD | Q51073 | Vp_VmeB | Q2AAU3 | Ea_AcrB | E3DBE3 |
| Pa_MexB | P52002 | Pa_TriC | Q9I6X4 | Kp_AcrB | Q93K40 |
| Ec_AcrD | P24177 | Ft_AcrB | A0Q8A5 | Hi_AcrB | Q57124 |
| Pp_ArpB | Q9KJC2 | Ab_AdeJ | Q24LT7 | Bp_BpeF | Q63NK6 |
| Pp_TtgB | O52248 | Vc_VexF | A6P7H3 | | |
| Pp_TtgE | Q9KWV4 | Smr_SdeB | Q84GI9 | | |
| Pp_TtgH | Q93PU4 | Pa_MexI | Q9HWH4 | | |
| Ec_MdtB | P76398 | Pa_MexW | A0A6A9K223 | | |
| Ec_MdtC | P76399 | Pa_MexQ | Q4LDT6 | | |
| Ec_MdtF | P37637 | Pa_MexN | Q4LDT8 | | |
| Sm_SmeW | B2FLY4 | Vc_VexB | Q9KVI2 | | |
| Pa_MexD | Q51396 | Vc_VexD | A6P7H1 | | |
| Pa_MexF | Q9I0Y8 | Vc_VexK | Q9KRG9 | | |
| Pa_MexK | Q9HXW4 | Pa_MuxC | Q9I0V7 | | |
| Pf_EmhB | Q6V6X8 | Pa_MuxB | Q9I0V6 | | |
| Ka_EefB | Q8GC83 | Ab_AdeB | Q2FD70 | | |
| Bg_ToxH | Q4VSJ4 | Sm_SmeB | Q9RBY8 | | |
| Pa_MexY | Q9ZNG8 | Sm_SmeE | Q9F240 | | |
| Cj_CmeB | Q8RTE4 | Sm_SmeJ | A0A0U5D5D4 | | |

**Supplementary Table 2: AcrB substrates for the phenotype assays.** The table lists the AcrB substrates used in the plate dilution assays (PDA) and for minimal inhibitory concentration (MIC) determination. The substrate class, the abbreviation used in the figures and the concentration used in the PDAs are indicated.

| **Substrate** | **Class** | **Abbreviation** | **Concentration [µg/mL]** |
|---|---|---|---|
| Chloramphenicol | Phenicol | CAM | 1.5 |
| Thiamphenicol | Phenicol | TIA | 12 |
| Linezolid | Oxazolidinone | LIN | 20 |
| Tetracycline | Tetracycline | TET | 0.3 |
| Oxytetracycline | Tetracycline | OxTET | 0.25 |
| Tigecycline | Tetracycline | TIG | 0.125 |
| Chlortetracycline | Tetracycline | ClTET | 0.2 |
| Doxycycline | Tetracycline | DXC | 0.5 |
| Minocycline | Tetracycline | MIN | 0.3 |
| Dicloxacillin | Beta-Lactam (Penicillin) | DIC | 36 |
| Oxacillin | Beta-Lactam (Penicillin) | OXA | 16 |
| Piperacillin | Beta-Lactam (Penicillin) | PIP | 0.075 |
| Dodecyl-β-D-maltosid | Detergent | DDM | 75 |
| Sodium dodecyl sulfate | Detergent | SDS | 70 |
| Erythromycin | Macrolide | ERY | 20 |
| Clarythromycin | Macrolide | CLA | - |
| Novobiocin | Aminocoumarin | NOV | 10 |
| Fusidic acid | Steroid | FUA | 12 |
| Doxorubicin | Anthracycline | DOX | 40 |
| Hoechst33342 | Dye | H33342 | 5 |
| Tetraphenylphosphonium | Phosphonium cation | TPP | 300 |
| Berberine | Dye | BER | - |

**Supplementary Table 3: Minimal inhibitory concentration (MIC) values.** BW25133 Δ*acrB* cells expressing AcrB wildtype (wt) or V612 variants were exposed to a serial dilution of a toxic substrate. The inactive D407N was used as negative control. The MIC value corresponds to the first dilution step at which no growth was detected. The table shows the mean MIC value for at least three biological replicates in µg/mL.

| | **wt** | **D407N** | **V612F** | **V612W** | **V612N** | **V612A** |
|---|---|---|---|---|---|---|
| **Chloramphenicol** | 4 | 1-2 | 8 | 8 | 8 | 8 |
| **Thiamphenicol** | 32 | 16 | 64 | 32-64 | 64 | 32 |
| **Linezolid** | 128 | 8 | 256 | 256 | 128-256 | 256 |
| **Tetracycline** | 1 | 0.25-0.5 | 1 | 1 | 1 | 1 |
| **Oxytetracycline** | 0.5 | 0.25 | 1 | 1 | 1 | 1 |
| **Tigecycline** | 0.25 | 0.125 | 0.125-0.25 | 0.25 | 0.25 | 0.25 |
| **Chlortetracycline** | 1 | 0.25 | 1 | 1 | 1 | 1 |
| **Doxycycline** | 2 | 0.25 | 2 | 2 | 2 | 2 |
| **Minocycline** | 4-16 | 0.25-8 | 0.5-2 | 16 | 16 | 16 |
| **Dicloxacillin** | 256 | 8-32 | 128 | 128 | 128 | 256 |
| **Oxacillin** | 128 | 2-8 | 64 | 64-128 | 64 | 128 |
| **Piperacillin** | 0.5 | 0.0625-0.125 | 0.125-0.25 | 0.25-0.5 | 0.25-0.5 | 0.5 |
| **SDS** | >8192 | 64 | 64 | 64-128 | >8192 | >8192 |
| **Erythromycin** | 128 | 8 | 64 | 64 | 64 | 64-128 |
| **Clarythromycin** | 64-128 | 4-16 | 32-64 | 64 | 32 | 64 |
| **Novobiocin** | 8-16 | 1-4 | 2-8 | 8 | 8 | 16-32 |
| **Fusidic acid** | 64-128 | 8-16 | 32-64 | 32-64 | 32 | 64 |
| **Hoechst33342** | 4-8 | 1-2 | 2-4 | 2 | 4 | 4 |

**Supplementary Table 4: Estimated binding free energies for the docking poses in Supplementary Figure 5.** ΔGb gives the total binding free energy in kcal/mol. The contributions from each individual residue involved in the interactions are indicated. Values are coloured from highest (white) to lowest (grey). Abbreviations: MIN – minocycline, DOX- doxorubicin, ERY – erythromycin, CAM – chloramphenicol.

**MIN**

| | ***ΔGb*** | ***S48*** | ***Q176*** | ***L177*** | ***F178*** | ***G179*** | ***S180*** | ***E273*** | ***N274*** | ***I277*** | ***A279*** | ***S287*** | ***F610*** | ***V/F/W612*** | ***F615*** | ***R620*** |
|---|---|---|---|---|---|---|---|---|---|---|---|---|---|---|---|---|
| **wt** | -28.2 | -0.7 | | -1.4 | -6.3 | -4.2 | -1.3 | -1.5 | -5.7 | -5.8 | -0.7 | -0.6 | -0.8 | -1.5 | -2.4 | -7.8 |
| **V612F** | -28.7 | -1.0 | -1.1 | -3.0 | -6.4 | -7.1 | -2.6 | | -4.3 | -5.2 | -1.0 | | -0.6 | -2.1 | -1.0 | |
| **V612W** | -25.6 | -3.5 | -0.7 | -1.8 | -5.8 | -4.0 | -2.3 | -1.3 | -5.3 | -5.0 | -0.7 | | | -2.3 | -1.5 | |

**DOX**

| | ***ΔGb*** | ***T44*** | ***S46*** | ***Q89*** | ***S128*** | ***E130*** | ***S132*** | ***S133*** | ***S134*** | ***F136*** | ***Q176*** | ***F178*** | ***G179*** | ***I277*** | ***F610*** | ***V/F/W612*** | ***F615*** | ***F617*** | ***R620*** | ***F628*** |
|---|---|---|---|---|---|---|---|---|---|---|---|---|---|---|---|---|---|---|---|---|
| **wt** | -53.5 | -1.1 | -5.9 | -3.8 | -6.7 | -7.8 | | | | -1.3 | -5.2 | -5.5 | | -1.9 | -1.0 | -2.0 | -2.3 | -0.7 | | |
| **V612F** | -46.4 | | -2.2 | -3.4 | -2.2 | -4.9 | | | -1.1 | | -5.8 | -7.8 | -1.2 | -3.4 | -0.8 | -4.0 | -3.1 | | -1.8 | -1.1 |
| **V612W** | -54.2 | -2.9 | | -5.1 | | -5.3 | -7.7 | -1.7 | -5.7 | -2.1 | -3.6 | -1.9 | | | | -3.5 | -5.9 | -1.0 | | |

**ERY**

| | ***ΔGb*** | ***T44*** | ***Q89*** | ***T91*** | ***E130*** | ***S132*** | ***S133*** | ***S134*** | ***F136*** | ***Q176*** | ***F178*** | ***K292*** | ***V/F/W612*** | ***F615*** | ***F617*** | ***R620*** | ***E673*** |
|---|---|---|---|---|---|---|---|---|---|---|---|---|---|---|---|---|---|
| **wt** | -50.3 | -2.0 | -7.0 | -2.4 | -2.5 | -7.7 | -2.4 | -12.1 | -2.2 | -3.8 | -1.7 | -5.7 | | -5.2 | -3.6 | -1.8 | -2.1 |
| **V612F** | -52.1 | -2.1 | -6.9 | -2.3 | -2.5 | -7.6 | -2.6 | -12.1 | -2.1 | -3.8 | -1.7 | -5.6 | -2.2 | -5.2 | -3.6 | -1.7 | -2.1 |
| **V612W** | -52.8 | -2.1 | -6.9 | -2.3 | -2.5 | -7.6 | -2.6 | -12.1 | -2.1 | -3.8 | -1.6 | -5.6 | -2.9 | -5.5 | -3.6 | -1.6 | -2.1 |

**CAM**

| | ***ΔGb*** | ***S134*** | ***F136*** | ***V139*** | ***Q151*** | ***Q176*** | ***L177*** | ***F178*** | ***G179*** | ***I277*** | ***I278*** | ***A279*** | ***P326*** | ***Y327*** | ***V571*** | ***M573*** | ***F610*** | ***V/F/W612*** | ***F615*** | ***F617*** | ***I626*** | ***F628*** | ***L668*** | ***V672*** |
|---|---|---|---|---|---|---|---|---|---|---|---|---|---|---|---|---|---|---|---|---|---|---|---|---|
| **wt** | -29.6 | -7.1 | -3.0 | | | | | | | | | | | -3.3 | -1.7 | -2.4 | | | -1.5 | -1.1 | -0.6 | -5.4 | -1.5 | -1.9 |
| **V612F** | -33.6 | | | | -1.4 | -4.6 | -3.7 | -6.1 | -2.8 | -4.9 | -0.8 | -1.6 | | | | | -0.7 | -3.6 | -1.8 | | -0.8 | -1.0 | | |
| **V612W** | -30.6 | | | -1.4 | | | | -2.3 | | -1.4 | | | -0.9 | -1.5 | | -0.8 | | -8.1 | -5.3 | -1.4 | -1.4 | -4.7 | | |

**Supplementary Table 5: Structural comparison of the TTO structure of OqxB (PDB ID: 8zxs, this study) with the TTT structure of OqxB (PDB ID: 7cz9) and LTO structure of AcrB (PDB ID: 4dx5)**

| | | AcrB (4dx5) | | | OqxB (7cz9) | | |
|---|---|---|---|---|---|---|---|
| | | Chain A (L) | Chain B (T) | Chain C (O) | Chain A (T) | Chain B (T) | Chain C (T) |
| OqxB (8zxs) | Chain A | 2.95 | 2.81 | 3.50 | 0.81 | 1.06 | 0.89 |
| | Chain B | 3.27 | 2.69 | 3.49 | 0.85 | 0.92 | 0.99 |
| | Chain C | 3.72 | 3.77 | 2.39 | 2.51 | 2.40 | 2.49 |

The RMSD (root mean square deviation) in Å is calculated between aligned pairs of the backbone Cα atoms in each monomer by LSQKAB in CCP4 program suite.

**Supplementary Table 6: Data collection and refinement statistics crystallographic structures AcrB V612W**

| | AcrB V612W MIY (TTT)<br>PDB ID: 9FE2 | AcrB V612W apo (TTT)<br>PDB ID: 9FE3 |
|---|---|---|
| **Data collection** | | |
| Space group | I 2 3 | I 2 3 |
| Cell dimensions | | |
| *a*, *b*, *c* (Å) | 227.49, 227.49, 227.49 | 227.43, 227.43, 227.43 |
| α, β, γ (°) | 90.00, 90.00, 90.00 | 90.00, 90.00, 90.00 |
| Resolution (Å) | 46.44 - 1.89 (1.96 - 1.89) | 44.60 - 2.30 (2.38 - 2.30) |
| $R_{sym}$ or $R_{merge}$ | 0.01656 (1.971) | 0.01593 (2.029) |
| $I / \sigma I$ | 21.91 (0.32) | 19.98 (0.33) |
| Completeness (%) | 96.99 (70.06) | 97.68 (77.33) |
| Redundancy | 2.0 (2.0) | 2.0 (2.0) |
| | | |
| **Refinement** | | |
| Resolution (Å) | 46.44 - 1.89 (1.96 - 1.89) | 44.60 - 2.3 (2.38 - 2.3) |
| No. reflections | 150429 (10838) | 84315 (6626) |
| $R_{work}$ / $R_{free}$ | 0.2239 / 0.2455 | 0.2535 / 0.2909 |
| No. atoms | | |
| Protein | 9499 | 9073 |
| Ligand/ion | 33 | 0 |
| Water | 384 | 6 |
| *B*-factors | | |
| Protein | 56.84 | 97.59 |
| Ligand/ion | 83.56 | - |
| Water | 53.54 | 65.66 |
| R.m.s. deviations | | |
| Bond lengths (Å) | 0.012 | 0.003 |
| Bond angles (°) | 1.19 | 0.65 |

*Values in parentheses are for highest-resolution shell.

**Supplementary Table 7: Data collection and refinement statistics crystallographic structures AcrB V612F**

| | AcrB V612F MIY (TTT)<br>PDB ID: 9FHC | AcrB V612F apo (TTT)<br>PDB ID: 9FE4 |
|---|---|---|
| **Data collection** | | |
| Space group | I 2 3 | P 3 2 1 |
| Cell dimensions | | |
| *a*, *b*, *c* (Å) | 227.46, 227.46, 227.46 | 134.41, 134.41, 190.97 |
| α, β, γ (°) | 90.00, 90.00, 90.00 | 90.00, 90.00, 120.00 |
| Resolution (Å) | 29.87 - 2.20 (2.279 - 2.20) | 49.70 - 2.80 (2.90 - 2.80) |
| $R_{sym}$ or $R_{merge}$ | 0.1293 (0.9247) | 0.09319 (3.095) |
| $I / \sigma I$ | 27.16 (4.31) | 20.19 (0.90) |
| Completeness (%) | 94.51 (67.04) | 99.73 (99.79) |
| Redundancy | 24.9 (21.4) | 11.0 (10.3) |
| | | |
| **Refinement** | | |
| Resolution (Å) | 29.87 - 2.20 (2.279 - 2.20) | 49.70 - 2.80 (2.90 - 2.80) |
| No. reflections | 93142 (6569) | 49611 (4859) |
| $R_{work}$ / $R_{free}$ | 0.2080 / 0.2380 | 0.2696 / 0.3030 |
| No. atoms | | |
| Protein | 9115 | 7843 |
| Ligand/ion | 33 | 0 |
| Water | 281 | 0 |
| *B*-factors | | |
| Protein | 40.76 | 110.83 |
| Ligand/ion | 39.25 | - |
| Water | 33.70 | - |
| R.m.s. deviations | | |
| Bond lengths (Å) | 0.008 | 0.009 |
| Bond angles (°) | 1.27 | 1.21 |

*Values in parentheses are for highest-resolution shell.

**Supplementary Table 8: Data collection and refinement statistics crystallographic structures AcrB V612N**

| | AcrB V612N (LTO)<br>PDB ID: 9FHG | AcrB V612N (TTT)<br>PDB ID: 9FHJ |
|---|---|---|
| **Data collection** | | |
| Space group | P 21 21 21 | I 2 3 |
| Cell dimensions | | |
| *a*, *b*, *c* (Å) | 145.81, 161.40, 245.41 | 228.65, 228.65, 228.65 |
| α, β, γ (°) | 90.00, 90.00, 90.00 | 90.00, 90.00, 90.00 |
| Resolution (Å) | 49.44 - 3.00 (3.11 - 3.00) | 41.74 - 3.55 (3.68 - 3.55) |
| $R_{sym}$ or $R_{merge}$ | 0.06477 (0.8325) | 0.01899 (2.092) |
| $I / \sigma I$ | 6.86 (1.03) | 15.67 (0.33) |
| Completeness (%) | 99.75 (99.71) | 93.89 (45.09) |
| Redundancy | 2.0 (2.0) | 2.0 (2.0) |
| | | |
| **Refinement** | | |
| Resolution (Å) | 49.44 - 3.00 (3.11 - 3.00) | 41.74 - 3.55 (3.68 - 3.55) |
| No. reflections | 115982 (11502) | 22677 (1078) |
| $R_{work}$ / $R_{free}$ | 0.2239 / 0.2829 | 0.2719 / 0.3260 |
| No. atoms | | |
| Protein | 25972 | 9086 |
| Ligand/ion | 0 | 0 |
| Water | 0 | 0 |
| *B*-factors | | |
| Protein | 79.72 | 195.91 |
| Ligand/ion | - | - |
| Water | - | - |
| R.m.s. deviations | | |
| Bond lengths (Å) | 0.009 | 0.004 |
| Bond angles (°) | 1.14 | 0.68 |

*Values in parentheses are for highest-resolution shell.

**Supplementary Table 9: Data collection and refinement statistics crystallographic structure OqxB**

| | OqxB (TTO)<br>PDB ID: 8ZXS |
|---|---|
| **Data collection** | |
| Space group | P 21 21 21 |
| Cell dimensions | |
| *a*, *b*, *c* (Å) | 121.39, 165.94, 249.03 |
| α, β, γ (°) | 90.00, 90.00, 90.00 |
| Resolution (Å) | 49.34 - 2.75 (2.80 - 2.75) |
| $R_{sym}$ or $R_{merge}$ | 0.082 (>1.0) |
| $I / \sigma I$ | 12.9 (0.9) |
| Completeness (%) | 99.65 (95.19) |
| Redundancy | 7.33 (7.30) |
| | |
| **Refinement** | |
| Resolution (Å) | 49.34 - 2.75 |
| No. reflections | 130594 (6157) |
| $R_{work}$ / $R_{free}$ | 0.2363 / 0.2877 |
| No. atoms | |
| Protein | |
| Ligand/ion | |
| Water | |
| *B*-factors | 124.7 |
| Protein | |
| Ligand/ion | |
| Water | |
| R.m.s. deviations | |
| Bond lengths (Å) | 0.005 |
| Bond angles (°) | 0.888 |

*Values in parentheses are for highest-resolution shell.

**Supplementary Table 10: Cryo-EM data collection, refinement and validation statistics**

| | OqxB (O*O*O*)<br>EMD-50334<br>PDB ID: 9FDZ | AcrB V612F (O)<br>EMD-50332<br>PDB ID: 9FDQ | AcrB V612W (O)<br>EMD-50331<br>PDB ID: 9FDP |
|---|---|---|---|
| **Data collection and processing** | | | |
| Magnification | 105000 | 130000 | 105000 |
| Voltage (kV) | 300 | 300 | 300 |
| Electron exposure (e–/Å$^2$) | 50 | 60 | 50 |
| Defocus range (μm) | -0.8 to -2.4 | -0.5 to -3.0 | -0.8 to -3.5 |
| Pixel size (Å) | 0.837 | 0.68 | 0.837 |
| Symmetry imposed | no | no | no |
| Initial particle images (no.) | 2703412 | 732894 | 856118 |
| Final particle images (no.) | 543928 | 120564 | 81754 |
| Map resolution (Å) | 2.86 | 3.47 | 3.3 |
| FSC threshold | 0.143 | 0.143 | 0.143 |
| | | | |
| **Refinement** | | | |
| Initial model used (PDB code) | AlphaFold | 4dx5 | 4dx5 |
| Model resolution (Å) | 2.86 | 3.4 | 3.3 |
| FSC threshold | 0.143 | 0.143 | 0.143 |
| Map sharpening *B* factor (Å$^2$) | - | - | - |
| Model composition | | | |
| Non-hydrogen atoms | 23736 | 7853 | 7857 |
| Protein residues | 3120 | 1033 | 1033 |
| Ligands | 0 | 0 | 0 |
| *B* factors (Å$^2$) | | | |
| Protein | 49.18/217.07/114.41 | 14.15/112.77/38.75 | 14.15/112.77/38.75 |
| Ligand | - | - | - |
| R.m.s. deviations | | | |
| Bond lengths (Å) | 0.004 | 0.002 | 0.002 |
| Bond angles (°) | 0.951 | 0.549 | 0.507 |
| Validation | | | |
| MolProbity score | 1.10 | 1.18 | 1.15 |
| Clashscore | 3.10 | 3.91 | 3.59 |
| Poor rotamers (%) | 0.59 | 0.00 | 0.00 |
| Ramachandran plot | | | |
| Favored (%) | 98.78 | 99.22 | 99.32 |
| Allowed (%) | 1.22 | 0.78 | 0.68 |
| Disallowed (%) | 0.00 | 0.00 | 0.00 |

## Supplementary Notes

# Conformational plasticity across phylogenetic clusters of RND multidrug efflux pumps and its impact on substrate specificity

Mariya Lazarova[1], Thomas Eicher[1], Clara Börnsen[2], Hui Zeng[1], Mohd Athar[3], Ui Okada[4], Eiki Yamashita[5], Inga M. Spannaus[1], Max Borgosch[1], Hi-jea Cha[1], Attilio V. Vargiu[3], Satoshi Murakami[4*], Kay Diederichs[6*], Achilleas S. Frangakis[2*], Klaas M. Pos [1*]

[1] Institute of Biochemistry, Goethe-University Frankfurt, Germany

[2] Buchmann Institute for Molecular Life Sciences and Institute of Biophysics, Goethe-University Frankfurt, Germany

[3] Department of Physics, University of Cagliari, Italy

[4] Department of Life Science and Technology, Tokyo Institute of Technology, Yokohama, Japan

[5] Institute for Protein Research, Osaka University, Japan

[6] Department of Biology, University of Konstanz, Germany

** Corresponding authors*

Klaas M. Pos, pos@em.uni-frankfurt.de

Achilleas S. Frangakis, achilleas.frangakis@biophysik.org

Kay Diederichs, kay.diederichs@uni-konstanz.de

Satoshi Murakami, murakami@bio.titech.ac.jp

**Homology modelling, ensemble-docking, molecular dynamics (MD) simulations, and free energy of binding calculations**

Despite our efforts to determine experimental co-structures with different AcrB substrates bound to the V612F/W variants, we only obtained co-structures with minocycline. To assess changes in the DBP interactions for further substrates, we therefore performed a computational study, namely docking, MD simulations, and free energy of binding calculations. For our study we choose four established AcrB substrates: minocycline, doxorubicin, erythromycin and chloramphenicol. For minocycline experimental co-structures are present for the wildtype (PDB ID: 4dx5) and the V612F/W variants (this study). This allows us to evaluate how the experimental data correlate with the computational study. For doxorubicin, an experimental co-structure is present for the wildtype (PDB ID: 4dx7) which also gives us a cross-reference for the computed results (Supplementary Notes Figure 1). Doxorubicin is an anthracycline and its chemical structure and binding site within AcrB (deep binding pocket, DBP) is very similar to the tetracycline antibiotics. However, in contrast to the tetracyclines, doxorubicin shows much higher reduction in the resistance activity of the variants. Further, there is a distinct phenotype discrepancy between the V612F and V612W variants (Fig. 1 main manuscript). For erythromycin and chloramphenicol (Supplementary Notes Figure 2 and 3), the V612F/W substitution induced changes of the phenotype mimicking that of the MdtF variant and of the proteins from the OqxB cluster, which contain an F at the position equivalent to V612 [4–8,18].

The top docking binding poses of minocycline closely resemble the experimental structures (Supplementary Figure 6). Moreover, the results of the free energy of binding calculations suggest that minocycline is stabilised by a network of interactions involving the hydrophilic groups of the ligand and the polar side chains or backbone carbonyl and amide groups of S48, L177, G179, S180, N274, I277, and A279 (Supplementary Table 4). F178, F610, F615 and the F/W612 also contribute to the interaction. In wildtype AcrB, R620 greatly contributes to the free binding energy, but this interaction is lost in the V612F and V612W variants as observed also in the experimental structures. However, this is compensated by a higher contribution of the substituted F/W612, as well as further individual side chains (L177, G179, S180 for V612F and S48, S148 for V612W). The total free energy of binding for the variants is similar to the wildtype with a difference of only 0.5 kcal/mol for V612F and 2.6 kcal/mol for V612W. Thus, the calculated binding poses and the experimental data both suggest the same mode of minocycline binding with hydrophilic interactions between the polar groups of the ligand and polar residues and the backbone of AcrB, and coordination of the aromatic ring of minocycline between F178, F615 and F/W612. In agreement with the experimental data, the docking

calculations confirm that the loss or decrease of the individual interactions due to the shift in the binding position of minocycline in V612F/W are readily compensated by alternative interactions with further residues in the binding site. MD simulations were also performed starting from the top docking pose of minocycline bound to the wild-type and to the F/W612 variants. The results, obtained from three independent simulation replicas for each system, demonstrated stable binding behavior with minimal conformational drift (RMSD ~2–3 Å) (Supplementary Notes Figure 4). These findings support the optimal accommodation of the tetracyclic core within the binding pocket, consistent with observations from experimental structural data (Supplementary Notes Figure 5a and 6).

The top binding pose calculated for doxorubicin in the DBP of wildtype AcrB closely resembles the orientation observed in the previously published experimental structure of the wildtype [19] (Supplementary Figure 6) despite a slight (~1.5 Å) sliding back towards the entrance of the DBP. A similar but much more pronounced shift (> 4.5 Å) is observed for doxorubicin in V612W. In V612F, the calculated pose of the ligand is slightly tilted compared to the experimental structure. The differences in the orientation of V612F compared to the wildtype also induces changes in the coordination network, and many of the interactions observed for the wildtype are weakened or abolished in V612F (e.g. with T44, S46, S128, E130, F136) (Supplementary Table 4). Even though this is compensated by stronger or additional interactions with S134, F178, G179, I277, F612, F615, R620 and F628, the total free energy of binding for V612F is 7.1 kcal/mol higher than the wildtype. For V612W, the interactions are weaker compared to the wildtype for the same amino acid sidechains as for V612F. In contrast to V612F, for V612W this is compensated mainly by polar interactions with the serine-rich loop (S132, S133, S134), and with T44 and Q89, owned to the shift in the position of the ligand in the V612W structure. The difference in the total binding energy for V612W compared to the wildtype (0.7 kcal/mol) is much lower than for V612F (Supplementary Table 4).

Similarly to minocycline, in the docking poses for doxorubicin a sliding of the ligand is observed for V612W and indeed the overlay with the experimental doxorubicin structure shows a clear steric overlap of the ligand with the W612 side chain (Supplementary Figure 6). However, in the V612F docking results, such shift in the ligand position is not observed. Instead, here the F612 side chain is flipped away from the ligand binding site. As it can be seen from the overlay of the experimental V612F structure with the doxorubicin docking pose, F612 in the experimental structure would clash with the ligand (Supplementary Notes Figure 1). The

difference in the orientations of F612 in the experimental and docking structures might represent alternative conformations that F612, and potentially W612, can adopt.

The experimental and computational results for the binding of minocycline and the computational results for the binding of doxorubicin suggest that the steric hindrance introduced by the V612F/W substitution results in a sliding of the substrate towards the entrance of the DBP. Besides minocycline, one further representative of the tetracycline class, doxycycline, was found to bind in the same position in the DBP [1] and it is feasible that other tetracyclines bind in a similar fashion as well. Thus, it is likely that similar sliding of the drug occurs, but the versatility of the DBP presumably allows accommodation of the substrate and formation of an alternative interaction network, explaining the marginal change in the resistance phenotype of V612F/W against minocycline and other tetracyclines (see Fig. 1 main manuscript).

Despite the discussed differences in the binding poses of minocycline and doxorubicin, their binding sites are very similar in AcrB wildtype, V612F and V612W (Supplementary Figure 6). The V612F variant exhibits a significant reduction in resistance to doxorubicin compared to a milder reduction in resistance to minocycline. Doxorubicin (as a dimer) initially binds to the AP of the L state, as shown by Eicher et al. [19] . A similar hypothesis as for erythromycin suggests that the considerable reduction in doxorubicin efflux might also be a consequence of the reduction in L states in the V612F variant.

In contrast to the similar binding poses of minocycline and doxorubicin in the AcrB wildtype and V612F/W variants, the top binding poses of chloramphenicol differ in the three proteins (Supplementary Notes Figure 2a). Chloramphenicol is one of the smallest AcrB ligands and with a van-der-Waals volume of 249 $Å^3$ (calculated with Chemicalize, https://chemicalize.com/) it is almost 15x smaller than the volume of the DBP (approximately 3700 $Å^3$ [20]). The ligand is small, flexible and amphipathic, and it is likely that it can be coordinated in different grooves within the DBP. A cryo-EM structure of AcrB in the presence of chloramphenicol showing a density for the ligand in the DBP has been reported [21]. An overlay of the electron density map of this structure with the docking poses for the wildtype, V612F and V612W shows that the putative ligand density is in proximity of the docking pose for wildtype AcrB (Supplementary Notes Figure 2b). Earlier studies have shown that chloramphenicol frequently flips in the DBP of the T state [21,22], suggesting that this substrate can be accommodated in different poses and frequently changes between them. Our MD simulations of chloramphenicol further elucidate its dynamic behaviour in wt and AcrB variants. Consistent with these prior reports, this substrate exhibited a highly dynamic

behaviour, particularly in V612F (the average RMSD values from the docking pose amount to ~4 Å in the wildtype and ~7 Å in the AcrB variants) (Supplementary Notes Figure 4). In V612F and to a lesser extent in V612W, chloramphenicol migrated towards the groove of the DBP, from which a facilitated efflux could occur in view of the already discussed small size of this ligand (Supplementary Notes Figure 6a). While chloramphenicol remained stable at the cryo-EM density site in WT AcrB [10], it shifted significantly toward the exit gate in the V612F variant, with a lesser shift in V612W. Despite its dynamic behavior, chloramphenicol maintained most interactions with key residues such as F136, V139, F178 F610, W/F612 and F628 in AcrB mutants (Supplementary Notes Figure 6b and 7).

The different computational poses might indicate distinct binding sites of chloramphenicol within the DBP. The reason why one of these binding sites is preferred in the wildtype and the others in the variants, might be connected to the introduced substitution. In both V612F and V612W, the ligand is pulled up in the DBP and oriented so that the aromatic ring of chloramphenicol is facing the F/W612 side chain. Particularly in V612W it is well evident how the aromatic ring of the ligand is sandwiched between F178, F615 and W612 and these interactions likely stabilise the binding at this position. In the F612 variant the ligand similarly engages in aromatic interactions with F178 and F612 (Supplementary Figure 6 and Supplementary Table 4).

For chloramphenicol, a slight increase in the resistance conferred by V612F and V612W was observed (see Fig. 1 main manuscript). One can speculate that chloramphenicol can diffuse in and out of the AP and DBP while interacting with multiple low affinity binding sites in the PD. The additional aromatic interactions with F/W612 could stabilise the interactions with the hydrophobic cluster, pulling chloramphenicol further into the DBP and increasing its retention time, thus allowing a more efficient transport. Similar consideration might apply for further flexible, amphipathic, low molecular weight substrates that contain an aromatic ring. One AcrB substrate that fits this description is linezolid, and for this substrate a slight increase in the resistance conferred by V612F/W was observed as well.

Experimental co-structures of erythromycin show binding of the substrate at the interface between the AP and the DBP in the L state of *E. coli* AcrB [10,23,24]. In the close homolog AcrB from *K. pneumoniae* (96 % sequence similarity to *E. coli* AcrB), erythromycin binding within the DBP in the T state has been described [25] (Supplementary Notes Figure 3a). Presumably, this substrate initially binds in the L state and is guided to the interior of the porter domain during the transition from L to T. The docking pose of erythromycin in AcrB wildtype (T state) is in

closer proximity to the DBP compared to the experimental co-structure of erythromycin bound to the L state (Supplementary Notes Figure 3a). However, the docking position of erythromycin does not reach the same binding site as the one seen in the experimental structure of *K. pneumoniae* AcrB in the T state. As the calculated pose is located between both binding sites seen in the experimentally derived co-structures, it might represent an intermediate interaction mode along the way from the AP towards the DBP (Supplementary Notes Figure 3).

The docking pose of erythromycin is near the entrance of the DBP, but it does not reach the DBP groove in contrast to minocycline and doxorubicin (Supplementary Notes Figure 3b). The latter two bind deeply within the hydrophobic groove cluster and are in immediate vicinity of the residue at position 612. Erythromycin, however, is located further away from this residue. Therefore, the steric effects of the V612F/W substitution, that were observed for minocycline and doxorubicin, are likely less relevant for erythromycin. Further, erythromycin does not contain an aromatic ring for interaction with the introduced aromatic substitution like e.g. chloramphenicol. Considering the greater distance to and minor interactions with the residue at position 612, it is possible that the V612F/W substitutions have a less pronounced effect on the interaction network of erythromycin compared to the other investigated substrates. In agreement with these considerations, the docking calculations showed identical binding poses for erythromycin in AcrB wildtype, V612F and V612W and similar binding energies with only a minor difference due to the contribution from F/W612 (Supplementary Figure 6 and Supplementary Table 4).

As the experimental structures of erythromycin show binding of this substrate in the AP-DBP-interface in the L state, it is plausible that the drug is sequestered from the periplasm through the AP in this state. Likely the substrate is then guided to the PD interior during the transition from L to T and finally reached the DBP in the T state. Potentially, the initial interactions require the PD architecture in the L state and are less favourable if the AP adopts the T state structure, as proposed earlier [11]. In contrast to the wildtype, the V612 variants exhibits a structure with increased abundance of the T state at the expense of the L state. The reduced amount of L state monomers for initial binding might compromise the transport of substrates such as erythromycin, that require the interactions for initial uptake in this state.

**Supplementary Notes Table 1:** System setup details for all-atom MD simulations of AcrB in lipid bilayer (WT_CAM and WT_MIN)

| | | **WT_CAM** | **WT_MIN** |
|---|---|---|---|
| Simulation box dimensions | | 159.5 × 159.9 × 171.3 Å | 156.6 × 156.7 ×173.8 Å |
| Total number of atoms | all | 374,286 | 363501 |
| | protein | 47354 | 47354 |
| | lipids | 81,682 | 77662 |
| | water | 244,959 (81,653 water) | 238176 (79392 water) |
| | ions | 259 | 249 |
| | ligand | 32 | 60 |
| Salt concentration | | 0.15M | |
| Lipid composition | | 2:1 POPE:POPG | |
| Force Field | | AMBER ff19SB (protein), Lipid21 (lipids), OPC (water) | |
| Software | | AMBER24 | |

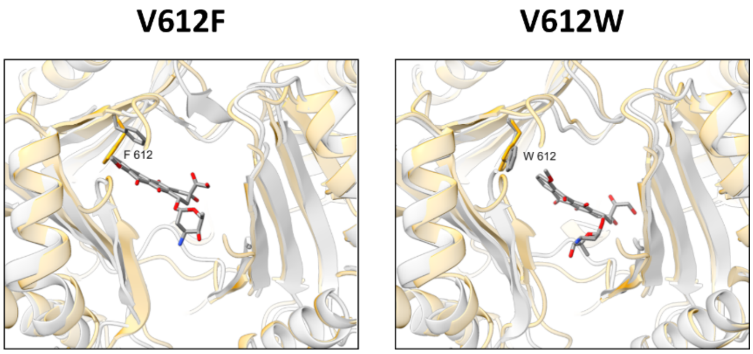


**Supplementary Notes Figure 1: Overlay of the experimental structures of AcrB V612F and V612W with the doxorubicin docking poses.** The figure shows the top docking pose of doxorubicin in the DBP of the V612F and V612W variants. AcrB is coloured grey with F/W612 and doxorubicin shown as sticks and coloured by atom type with carbon – grey, oxygen – red, and nitrogen – blue. The docking results are overlayed with the experimental crystallographic structures of AcrB V612F and V612W (this study) coloured in yellow.

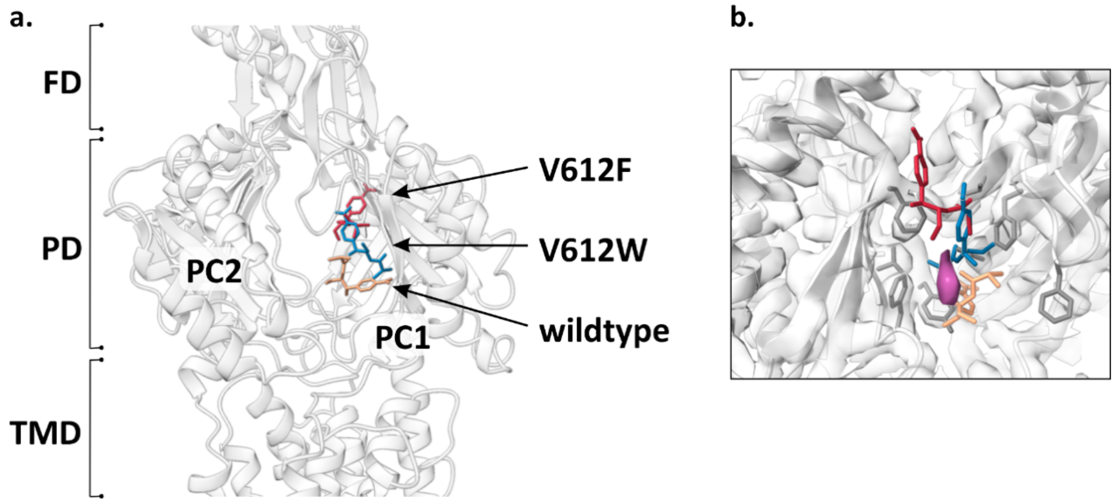


**Supplementary Notes Figure 2: Chloramphenicol binding in the DBP of AcrB.** (**a**) The top docking poses for chloramphenicol in the DBP of AcrB wildtype, V612F and V612W. The AcrB structure is outlined in the cartoon representation. Chloramphenicol docking poses are shown as sticks and coloured yellow for the wildtype, red for V612F and blue for V612W. Abbreviations: FD – funnel domain, PD – porter domain, TMD – transmembrane domain. The PC1 and PC2 subdomains are indicated. (**b**) Overlay of the top docking poses for chloramphenicol with the experimental AcrB wildtype structure in the presence of this substrate (PDB ID: 6sgr). The docking poses for the wildtype, V612F and V612W are shown as sticks coloured as in (a). The cryo-EM electron density map of the experimental structure is shown as surface with the putative chloramphenicol density highlighted in purple. The residues of the DBP are shown as sticks in grey.

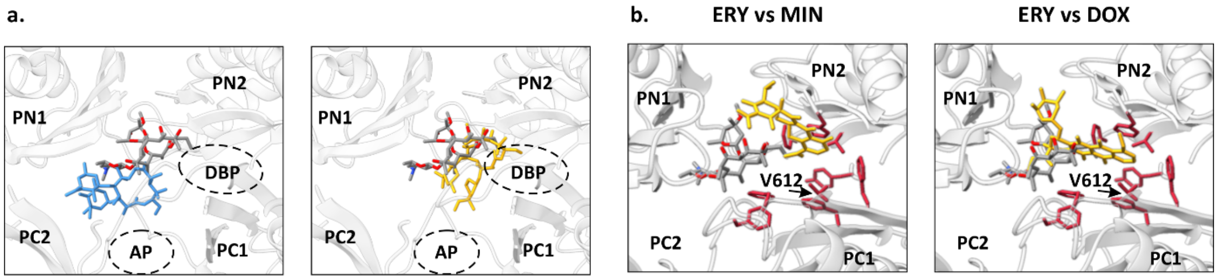


**Supplementary Notes Figure 3: Erythromycin binding sites in the PD of AcrB.** (**a**) In experimental co-structures of *E. coli* AcrB, erythromycin binds at the interface of the AP and DBP in the L state monomer (blue, left panel, PDB ID: 3aoc). In the close homolog AcrB from *K. pneumoniae*, the substrate was found in the DBP in the T state monomer (yellow, right panel, PDB ID: 8ffs). The top docking pose for AcrB wildtype (coloured by atom type with carbon – grey, oxygen – red, and nitrogen – blue) was overlayed with both structures. The PD is outlined as cartoon and the PN1, PN2, PC1 and PC2 subdomains as well as the AP and DBP are indicated. (**b**) An overlay of the docking pose of erythromycin with the minocycline (MIN, left panel) and doxorubicin (DOX, right panel) structures. A top view of the porter domain is shown in grey and the PN1, PN2, PC1 and PC2 subdomains are indicated. The residues of the DBP are shown as sticks in red and V612 is indicated. The top docking pose of erythromycin in wildtype AcrB is shown as sticks coloured by atom type with carbon – grey, oxygen – red, and nitrogen – blue. Minocycline and doxorubicin from the experimental structures (PDB ID: 4dx5 and 4dx7 respectively) are shown as sticks in yellow.

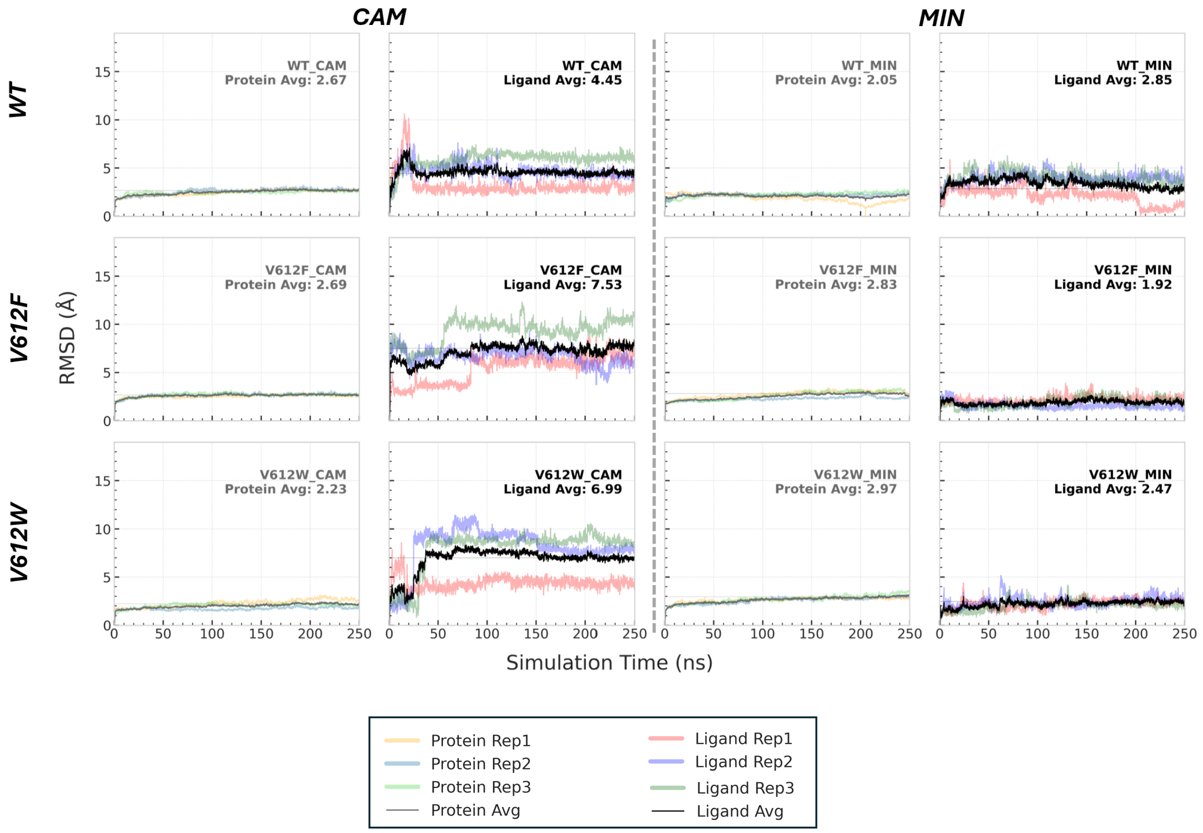


**Supplementary Notes Figure 4**: **RMSD profiles of protein backbone and ligand heavy atoms across six systems (WT, V612F, V612W bound to CAM or MIN)**. Three independent MD simulation replicas are shown, with individual RMSD traces displayed transparently and the average depicted as a solid line. For MIN-bound complexes, RMSD was calculated with respect to the corresponding experimental reference structures (V612F, V612W from this work, and for WT, PDB: 4dx5) using all heavy atoms. In contrast, CAM-bound complexes were evaluated against their initial docked conformations. Averages were calculated over the last 50 ns of the simulation for each system.

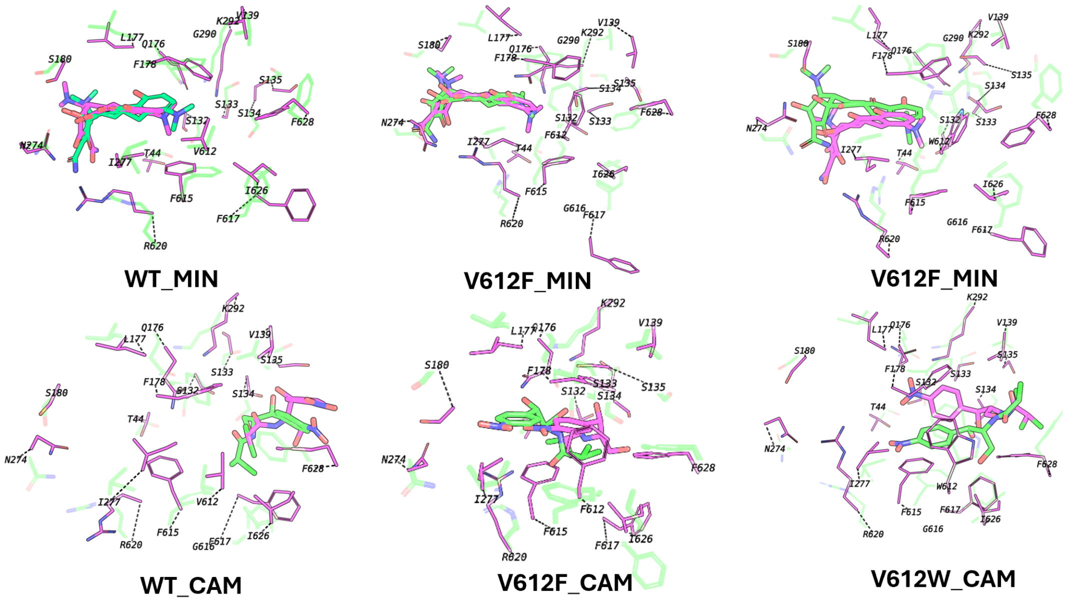


**Supplementary Notes Figure 5: Comparison between the experimental/docking structures (green) and the representative conformations (magenta).** The results were obtained from MD simulations (replica 1) of MIN and CAM in complex with wt and V612F/W variants. MIN and the DBP binding site residues are shown in green in the experimental structures (MIN bound to WT AcrB: PDB ID 4DX5; MIN bound to V612F and V612W variants: complexes from this work). The same representation is used to represent CAM-bound geometries obtained from docking. A representative structure obtained from MD simulations is shown in magenta sticks. Lines connect the Cα atoms of corresponding residues between the reference and the representative frame; the length of each line indicates the degree of positional shift, reflecting the extent of adaptive change during the simulation.

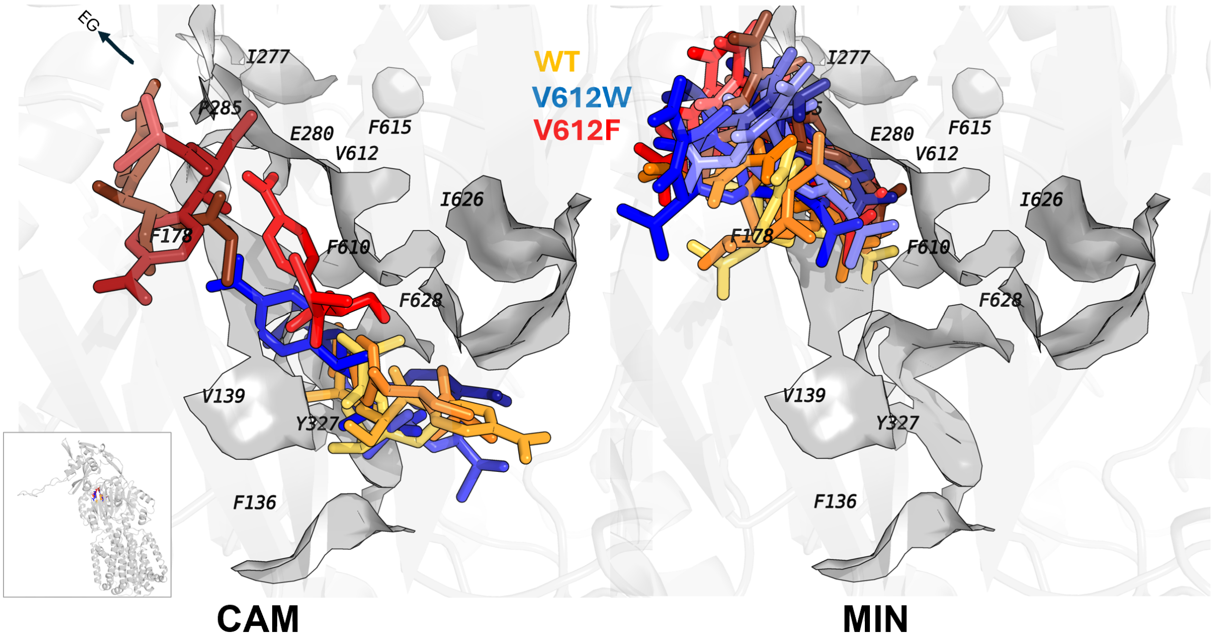


(a)

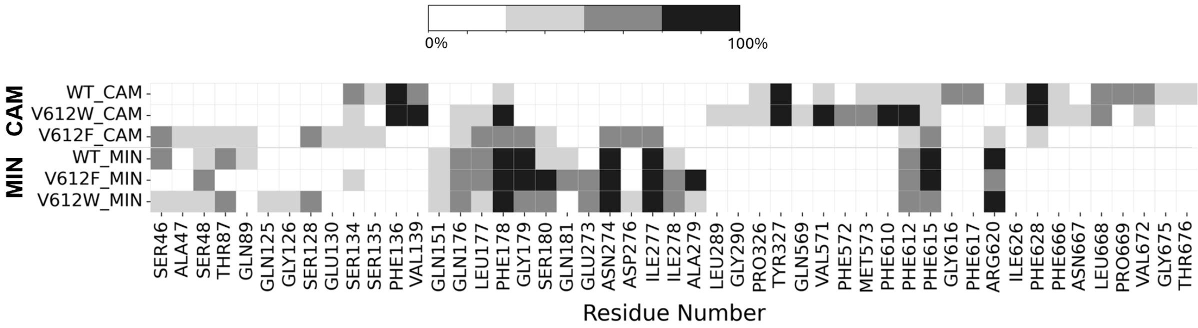


(b)

**Supplementary Notes Figure 6: MD poses of CAM and MIN and ligand–residue interaction summary heatmap. (a)** Representative MD poses of CAM and MIN with wt and AcrB variants derived from three independent replicas. The surface representation is generated from residue F136, V139, F178, I277, A279, E280, P285, Y327, F610, V612, F615, F617, I626, and F628 for the T-state [26]. The inset depicts the orientation of the AcrB T-monomer, with EG indicating the exit gate. MIN remains stably bound in the upper groove region, while CAM exhibits diverse orientations, progressively shifting upward toward the cave region from WT to V612W to V612F during the simulation. **(b)** Ligand–residue interaction summary heatmap. Contacts were calculated using Maestro Interaction Fingerprint tool on representative MD poses (obtained *via* hierarchical clustering, *see Methods*). For each ligand system, binary interaction data (presence or absence) were collected across three replicas and summarized to indicate interactions consistency with heatmap using grayscale colorbar ranging from 0% (white, absent in all replicas) to 100% (black, present in all three replicas).

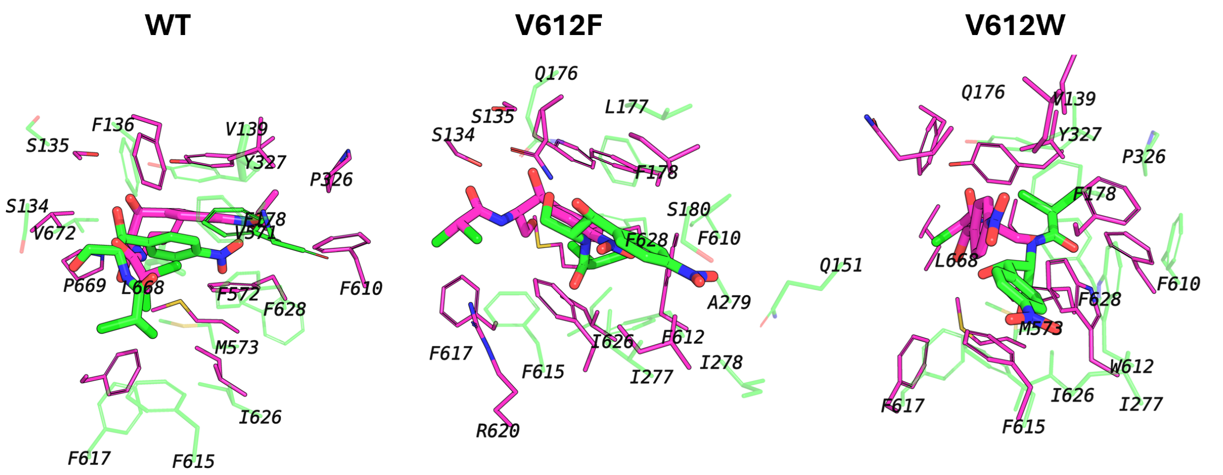


**Supplementary Notes Figure 7: Interactions within 4 Å of CAM in the initial (green carbons transparent) and MD-representative (magenta carbons solid) poses.** In the AcrB V612F/W mutants, CAM remains stably sandwiched between F178, F615, and F/W612, indicating persistent hydrophobic interactions. In contrast, the WT system shows a loss of F615 interaction in the MD-derived cluster.

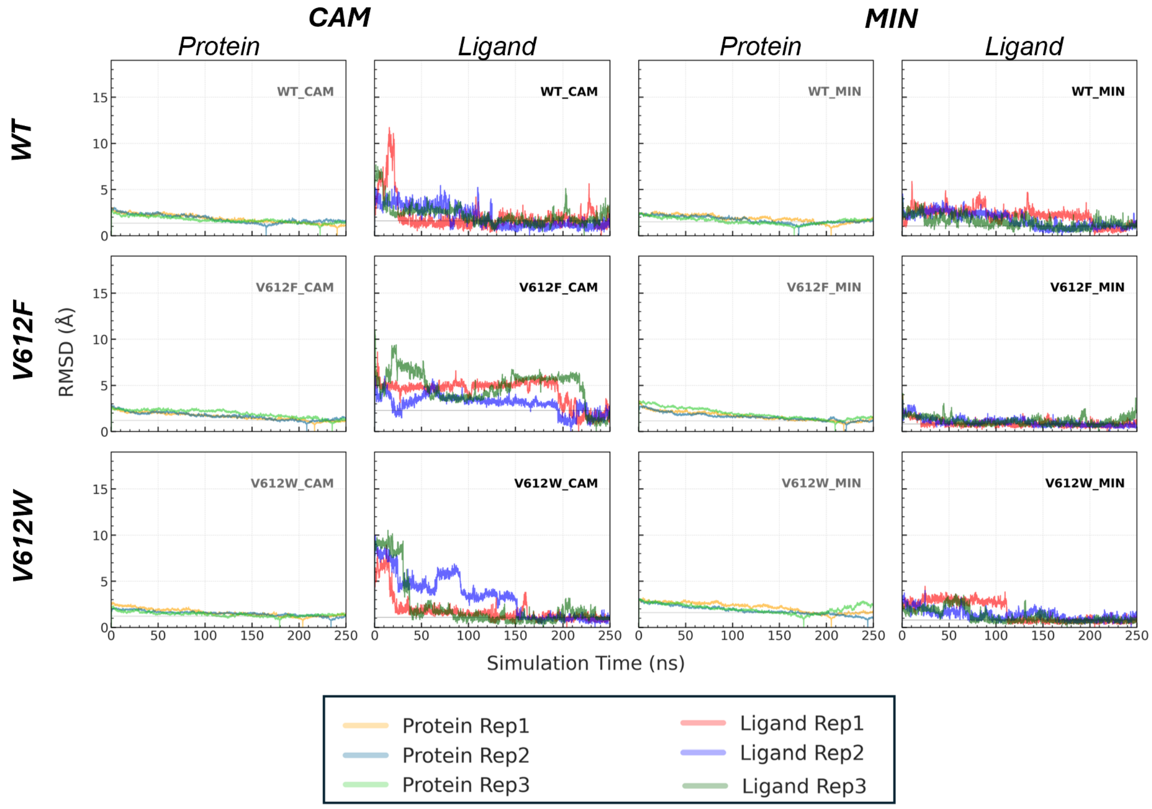


**Supplementary Notes Figure 8: Time-course analysis of equilibration for CAM and MIN systems across three MD replicas.** RMSD of the protein backbone and ligands (CLM, MYI) calculated from representative clusters extracted from the last 50 ns of 250-ns production runs. Protein backbone and ligand RMSD stayed below 2 Å, confirming system equilibration and convergence.

**Supplementary Methods**

# Conformational plasticity across phylogenetic clusters of RND multidrug efflux pumps and its impact on substrate specificity

Mariya Lazarova[1], Thomas Eicher[1], Clara Börnsen[2], Hui Zeng[1], Mohd Athar[3], Ui Okada[4], Eiki Yamashita[5], Inga M. Spannaus[1], Max Borgosch[1], Hi-jea Cha[1], Attilio V. Vargiu[3], Satoshi Murakami[4*], Kay Diederichs[6*], Achilleas S. Frangakis[2*], Klaas M. Pos [1*]

[1] Institute of Biochemistry, Goethe-University Frankfurt, Germany

[2] Buchmann Institute for Molecular Life Sciences and Institute of Biophysics, Goethe-University Frankfurt, Germany

[3] Department of Physics, University of Cagliari, Italy

[4] Department of Life Science and Technology, Tokyo Institute of Technology, Yokohama, Japan

[5] Institute for Protein Research, Osaka University, Japan

[6] Department of Biology, University of Konstanz, Germany

** Corresponding authors*

Klaas M. Pos, pos@em.uni-frankfurt.de

Achilleas S. Frangakis, achilleas.frangakis@biophysik.org

Kay Diederichs, kay.diederichs@uni-konstanz.de

Satoshi Murakami, murakami@bio.titech.ac.jp

Computational Methods

*Homology modelling, ensemble-docking, and free energy of binding calculations*

Ensemble-docking calculations were performed for all compounds on the DBP of the T state ($DBP_T$) of AcrB (lined by residues S46, Q89, S128, E130, S134, F136, V139, Q176, L177, F178, G179, S180, E273, N274, D276, I277, A279, A286, P326, Y327, M573, F610, V/F/W612, F617, R620, F628, F664, F666, L668, P669, V672 [27]) using the software GNINA v1.3 [28]. Following a previous approach [29,30], we generated a set of AcrB (wt, V612F, and V612W) structures featuring the largest structural variance at the $DBP_T$, in order to account for minor to medium structural changes at this site, which notoriously influence the outcome of docking calculations [31]. The list of AcrB structures we employed includes the following PDB IDs: 2dhh, 2dr6, 2drd, 2gif, 2hrt, 2j8s, 3aoa, 3aob, 3aoc, 3aod, 3noc, 3nog, 3w9h, 4dx5, 4dx6, 4dx7, 4u8v, 4u8y, 4u95, 4u96, 4zit, 4ziv, 4zjl, 5jmn, 5nc5, 5yil, 6q4n, 6q4o, 6q4p. In addition, for the V612F and the V612W AcrB variants, we included the new experimental structures reported here. Note that some of the afore listed structures contain gaps, modified residues, and mutations. Therefore, we generated consistent structures of the wt as well as the two variants of AcrB by means of homology modelling calculations, using the software MODELLER 10.2 [32]. More precisely, for the wt structures only the missing parts were modelled, using the wildtype structure (PDB ID: 4dx5) as a template. The models of the mutant structures were generated by modelling only the amino acids within 5 Å from the mutated residues, using as templates the experimental V612F and V612W structures. In both cases, the list of residues was the following: F178, I278, F610, A611, F/W612, N613, F615, G625, I626, F628. The rest of the structure was not modified, apart for gaps and additional mutations. Therefore, a multi-template homology modelling was performed for both the wt and the mutant structures. Moreover, we introduced the F/W612 mutations within the asymmetric LTO structures based on the TTT experimental geometries of V612F/W. Before running homology modelling calculations, all structures were aligned to the $DBP_T$ of the structure with PDB ID 4dx5, and only the amino acids from 1 to 1030 (in each chain) were used to ensure that all the models will have the same length.

After generating a pool of AcrB structures identical in sequence, we reassign protonation states of the protein following previous literature [20,33]. Next, we identified the T state in each structure and aligned all of them (29 for wt and 31 for mutants) to the $DBP_T$ of that identified by the PDB ID 4dx5 and calculated 29x29 (wt) and 31x31 (mutants) matrices of pairwise RMSDs values. From these matrices, we retained only the structures displaying RMSDs (calculated on all the

heavy atoms of $DBP_T$) larger than 1 Å from each other. For pairs with RMSDs values below this threshold, we removed the structure with the lowest resolution from the pool.

The following structure data files (*.sdf*) were used to prepare the pdb files for docking with GNINA: CAM and ERY were downloaded from our antibiotic database (structure optimized by means of quantum-mechanical calculations) [34], while MIN and DOX were taken from the PDB database (IDs 4dx5 and 4dx7, respectively). For each ligand, the dominant protonation states at physiological pH were assigned by ChemAxon's Marvin suite of program. Docking was performed centring the grid on $DBP_T$ and using the following parameters: exhaustiveness: 512 (default 8); nmodes: 10.

Poses were first filtered according to the Convolutional Neural Networks (CNN) score provided by GNINA (which ranges from 0 – very unlikely pose to 1 – very likely that the pose represents a true structure), retaining only those with a value larger than 2/3. Next, these poses were ranked according to their estimated affinity to the binding site.

Estimation of the binding free energy were obtained through the MM/GBSA approach [35] implemented in the MMPBSA.py tool of AMBER22 [36], following the same protocol used in previous studies [37,38]. This approach provides an intrinsically simple method for decomposing the free energy of binding into contributions from single atoms and residues, as well as ligand-residue pairs [39]. The solute conformational entropy contribution ($T\Delta S_{conf}$) was not evaluated [35]. Calculations were performed on the top pose of each complex.

In these methods [35, 40] the binding free energy of each compound is evaluated as:

$$\Delta G_{\text{bind}} = G_{\text{comp}} - \left(\Delta G_{\text{rec}} + G_{\text{lig}}\right)$$

with $G_{compl}$, $G_{rec}$, and $G_{lig}$ being the absolute free energies of complex, receptor, and ligand, respectively. According to these schemes, the free-energy difference can be also decomposed as

$$\Delta G_{\text{bind}} = \Delta E_{\text{MM}} + \Delta G_{\text{solv}} - T\Delta S_{\text{conf}}$$

where $\Delta E_{MM}$ is the difference in the molecular mechanics energy, $\Delta G_{solv}$ is the solvation-free energy, and $\Delta S_{conf}$ is the solute conformational entropy change. The first two terms were calculated with the following equations:

$$\Delta E_{\text{MM}} = \Delta E_{\text{bond}} + \Delta E_{\text{angle}} + \Delta E_{\text{torsion}} + \Delta E_{\text{vdw}} + \Delta E_{\text{elec}}$$

and

$$\Delta G_{\text{solv}} = \Delta G_{\text{solv,p}} + \Delta G_{\text{solv,np}}$$

$E_{MM}$ includes the molecular mechanics energy contributed by the bonded ($E_{bond}$, $E_{angle}$, and $E_{torsion}$) and nonbonded ($E_{vdw}$ and $E_{elec}$, calculated with no cutoff) terms of the force field. $\Delta G_{solv}$ is the solvation-free energy, which can be modeled as the sum of an electrostatic contribution ($\Delta G_{solv,p}$, evaluated using the MM-GBSA approach) and a nonpolar one ($\Delta G_{solv,np} = \gamma \Delta_{SA} + b$, proportional to the difference in solvent-exposed surface area, $\Delta_{SA}$).

Note that the solute entropy contributions were not calculated here, as they are notoriously challenging; thus, these values represent relative binding energies rather than absolute free energies.

*Molecular Dynamics Simulations*

The top docked poses of CAM and MIN with WT and V612F/W AcrB LTO trimers were used as starting structures for all-atom MD simulations. Protonation states were assigned according to the findings of Eicher *et al* [33], specifically, residues E346 and D924 were protonated only in L and T protomers, while residues D407, D408, and D566 were protonated only in the O protomer [41,42]. The *pdb4amber* command of AmberTools23 was used to assign GAFF2 atom types for ligands, remove existing hydrogens, and regenerate them as appropriate. Membrane embedding was done using PACKMOL-Memgen [43] placing the protein in a mixed symmetric bilayer composed of 1-palmitoyl-2-oleoyl-sn-glycero3-phosphoethanolamine (POPE) and 1-palmitoyl-2-oleoyl-sn-glycero-3-phosphoglycerol (POPG) in a 2:1 ratio across the two leaflets. Ligand parameters for CAM and MIN were taken from [34] . The ff19SB [44] forcefield was used for the protein, the lipid21 [45] for the lipids, and the OPC model [46] was used for water, coupled to ions parameters from [47] . Systems were solvated with OPC water layers (17.5 Å thickness) with 0.15 M KCl buffer solution (overall composition of the system shown in Supplementary Notes Table 1). Lipid packing was optimized with randomized placement followed by minimization. The system was constructed over 100 iterative cycles with a convergence criterion of 2.4 Å to ensure stability. Each system was first subjected to a multistep structural relaxation via a combination of steepest descent and conjugate gradient method using *pmemd.cuda* program implemented in AMBER24 as described previously [2,20,29,37,48,49]. The system was then heated in two stages: first, from 0 to 100 K over 1 ns under constant-volume conditions applying harmonic restraints (k = 1 $\text{kcalmol}^{-1}\text{Å}^{-2}$) to the heavy atoms of both the protein and the lipids, subsequently, temperature was increased from 100 to 310 K under constant pressure (1 atm) for 1 ns, using harmonic restraints (k = 2 $\text{kcal mol}^{-1}\text{ Å}^{-2}$) applied to the Cα atoms of the protein and *z* coordinates of the phosphorous atoms (P31) in lipids. This

approach allowed for membrane rearrangement during the heating process. The system was equilibrated in six consecutive steps at 1 fs time steps for 250 ps each (total 1.5 ns) with restraint on the protein coordinates under isotropic pressure scaling using the Berendsen barostat, whereas a Langevin thermostat (with a collision frequency of 1 $ps^{-1}$) was used to maintain a constant temperature. Time-course analysis confirmed equilibration, with temperature stabilizing at 310 K, water density reaching ~1 $g/cm^3$ after ~1.5 ns, and protein backbone RMSD remaining below 2 Å. Finally, three production simulations of 250 ns were performed at 310 K with a 2 fs time step employing Langevin thermostat and anisotropic pressure scaling (under an isothermal-isobaric ensemble), with outputs recorded every 200 ps. Initial structures for the production runs were selected by performing a cluster analysis (hierarchical agglomerative (average-linkage) in *cpptraj* on the cumulative equilibrated trajectory, using the ligands as selection for clustering after alignment of the T-state monomer of AcrB, and imposing a number of three clusters (equal to the number of production trajectories) as output. The Particle mesh Ewald algorithm was used to evaluate long-range electrostatic forces with a non-bonded cut-off of 9 Å. Trajectories post-processing was performed with *cpptraj* and equilibration of the system was verified as in Supplementary Notes Figure 8.

Representative conformations of each system from MD simulations were selected by performing a clustering on the last 50 ns of MD simulations, with frames pre-aligned to the binding site (defined as the list of residues within 4 Å of the ligand in the last 50 ns). Ligand RMSD (excluding hydrogens) was used to measure similarity without further alignment. Up to five clusters were generated, and the centroid of each was taken as the representative structure.

## Supplementary References


1. Ornik-Cha, A. *et al.* Structural and functional analysis of the promiscuous AcrB and AdeB efflux pumps suggests different drug binding mechanisms. *Nature communications* **12,** 6919; 10.1038/s41467-021-27146-2 (2021).

2. Plé, C. *et al.* Pyridylpiperazine-based allosteric inhibitors of RND-type multidrug efflux pumps. *Nature communications* **13,** 115; 10.1038/s41467-021-27726-2 (2022).

3. Zwama, M. *et al.* Multiple entry pathways within the efflux transporter AcrB contribute to multidrug recognition. *Nature communications* **9,** 124; 10.1038/s41467-017-02493-1 (2018).

4. Bohnert, J. A., Schuster, S., Fähnrich, E., Trittler, R. & Kern, W. V. Altered spectrum of multidrug resistance associated with a single point mutation in the Escherichia coli RND-type MDR efflux pump YhiV (MdtF). *The Journal of antimicrobial chemotherapy* **59,** 1216–1222; 10.1093/jac/dkl426 (2007).

5. Bharatham, N. *et al.* Structure and function relationship of OqxB efflux pump from Klebsiella pneumoniae. *Nature communications* **12,** 5400; 10.1038/s41467-021-25679-0 (2021).

6. Hansen, L. H., Jensen, L. B., Sørensen, H. I. & Sørensen, S. J. Substrate specificity of the OqxAB multidrug resistance pump in Escherichia coli and selected enteric bacteria. *The Journal of antimicrobial chemotherapy* **60,** 145–147; 10.1093/jac/dkm167 (2007).

7. Kato, T. *et al.* Crystal structures of multidrug efflux transporters from Burkholderia pseudomallei suggest details of transport mechanism. *Proceedings of the National Academy of Sciences of the United States of America* **120,** e2215072120; 10.1073/pnas.2215072120 (2023).

8. Coyne, S., Rosenfeld, N., Lambert, T., Courvalin, P. & Périchon, B. Overexpression of resistance-nodulation-cell division pump AdeFGH confers multidrug resistance in Acinetobacter baumannii. *Antimicrobial agents and chemotherapy* **54,** 4389–4393; 10.1128/AAC.00155-10 (2010).

9. Maseda, H., Yoneyama, H. & Nakae, T. Assignment of the substrate-selective subunits of the MexEF-OprN multidrug efflux pump of Pseudomonas aeruginosa. *Antimicrobial agents and chemotherapy* **44,** 658–664; 10.1128/AAC.44.3.658-664.2000 (2000).

10. Tam, H.-K. *et al.* Allosteric drug transport mechanism of multidrug transporter AcrB. *Nature communications* **12,** 3889; 10.1038/s41467-021-24151-3 (2021).

11. Seeger, M. A., Ballmoos, C. von, Verrey, F. & Pos, K. M. Crucial role of Asp408 in the proton translocation pathway of multidrug transporter AcrB: evidence from site-directed mutagenesis and carbodiimide labeling. *Biochemistry* **48,** 5801–5812; 10.1021/bi900446j (2009).

12. Scheres, S. H. W. RELION: implementation of a Bayesian approach to cryo-EM structure determination. *Journal of structural biology* **180,** 519–530; 10.1016/j.jsb.2012.09.006 (2012).

13. Punjani, A., Rubinstein, J. L., Fleet, D. J. & Brubaker, M. A. cryoSPARC: algorithms for rapid unsupervised cryo-EM structure determination. *Nature methods* **14,** 290–296; 10.1038/nmeth.4169 (2017).

14. Morgan, C. E. *et al.* Cryoelectron Microscopy Structures of AdeB Illuminate Mechanisms of Simultaneous Binding and Exporting of Substrates. *mBio* **12**; 10.1128/mbio.03690-20 (2021).

15. Su, C.-C. *et al.* Structures and transport dynamics of a Campylobacter jejuni multidrug efflux pump. *Nature communications* **8,** 171; 10.1038/s41467-017-00217-z (2017).

16. Zhang, Z. *et al.* Cryo-Electron Microscopy Structures of a Campylobacter Multidrug Efflux Pump Reveal a Novel Mechanism of Drug Recognition and Resistance. *Microbiology spectrum* **11,** e0119723; 10.1128/spectrum.01197-23 (2023).

17. Saier, M. H., Tran, C. V. & Barabote, R. D. TCDB: the Transporter Classification Database for membrane transport protein analyses and information. *Nucleic acids research* **34,** D181-6; 10.1093/nar/gkj001 (2006).

18. Köhler, T. *et al.* Characterization of MexE-MexF-OprN, a positively regulated multidrug efflux system of Pseudomonas aeruginosa. *Molecular microbiology* **23,** 345–354; 10.1046/j.1365-2958.1997.2281594.x (1997).

19. Eicher, T. *et al.* Transport of drugs by the multidrug transporter AcrB involves an access and a deep binding pocket that are separated by a switch-loop. *Proceedings of the National Academy of Sciences of the United States of America* **109,** 5687–5692; 10.1073/pnas.1114944109 (2012).

20. Ramaswamy, V. K., Vargiu, A. V., Malloci, G., Dreier, J. & Ruggerone, P. Molecular Rationale behind the Differential Substrate Specificity of Bacterial RND Multi-Drug Transporters. *Scientific reports* **7,** 8075; 10.1038/s41598-017-08747-8 (2017).

21. Du, D. *et al.* Interactions of a Bacterial RND Transporter with a Transmembrane Small Protein in a Lipid Environment. *Structure (London, England : 1993)* **28,** 625-634.e6; 10.1016/j.str.2020.03.013 (2020).

22. Vargiu, A. V. & Nikaido, H. Multidrug binding properties of the AcrB efflux pump characterized by molecular dynamics simulations. *Proceedings of the National Academy of Sciences of the United States of America* **109,** 20637–20642; 10.1073/pnas.1218348109 (2012).

23. Nakashima, R., Sakurai, K. & Yamaguchi, A. *Structures of the multidrug exporter AcrB reveal a proximal multisite drug-binding pocket* (2011).

24. Ababou, A. & Koronakis, V. Structures of Gate Loop Variants of the AcrB Drug Efflux Pump Bound by Erythromycin Substrate. *PloS one* **11,** e0159154; 10.1371/journal.pone.0159154 (2016).

25. Zhang, Z., Morgan, C. E., Bonomo, R. A. & Yu, E. W. Cryo-EM Structures of the Klebsiella pneumoniae AcrB Multidrug Efflux Pump. *mBio* **14,** e0065923; 10.1128/mbio.00659-23 (2023).

26. Takatsuka, Y., Chen, C. & Nikaido, H. Mechanism of recognition of compounds of diverse structures by the multidrug efflux pump AcrB of Escherichia coli. *Proceedings of the National Academy of Sciences of the United States of America* **107,** 6559–6565; 10.1073/pnas.1001460107 (2010).

27. Athar, M. *et al.* Tripartite efflux pumps of the RND superfamily: what did we learn from computational studies? *Microbiology (Reading, England)* **169**; 10.1099/mic.0.001307 (2023).

28. McNutt, A. T. *et al.* GNINA 1.0: molecular docking with deep learning. *Journal of cheminformatics* **13,** 43; 10.1186/s13321-021-00522-2 (2021).

29. Malvacio, I. *et al.* Molecular basis for the different interactions of congeneric substrates with the polyspecific transporter AcrB. *Biochimica et biophysica acta. Biomembranes* **1861,** 1397–1408; 10.1016/j.bbamem.2019.05.004 (2019).

30. Trampari, E. *et al.* Functionally distinct mutations within AcrB underpin antibiotic resistance in different lifestyles. *npj antimicrobials and resistance* **1,** 2; 10.1038/s44259-023-00001-8 (2023).

31. Basciu, A. *et al.* No dance, no partner! A tale of receptor flexibility in docking and virtual screening. In *Virtual Screening and Drug Docking* (Elsevier2022), Vol. 59, pp. 43–97.

32. Eswar, N. *et al.* Comparative protein structure modeling using Modeller. *Current protocols in bioinformatics* **Chapter 5,** Unit-5.6; 10.1002/0471250953.bi0506s15 (2006).

33. Eicher, T. *et al.* Coupling of remote alternating-access transport mechanisms for protons and substrates in the multidrug efflux pump AcrB. *eLife* **3**; 10.7554/eLife.03145 (2014).

34. Gervasoni, S. *et al.* AB-DB: Force-Field parameters, MD trajectories, QM-based data, and Descriptors of Antimicrobials. *Scientific data* **9,** 148; 10.1038/s41597-022-01261-1 (2022).

35. Genheden, S. & Ryde, U. The MM/PBSA and MM/GBSA methods to estimate ligand-binding affinities. *Expert opinion on drug discovery* **10,** 449–461; 10.1517/17460441.2015.1032936 (2015).

36. Case, D. A. *et al.* AmberTools. *Journal of chemical information and modeling* **63,** 6183–6191; 10.1021/acs.jcim.3c01153 (2023).

37. Vargiu, A. V., Ruggerone, P., Opperman, T. J., Nguyen, S. T. & Nikaido, H. Molecular mechanism of MBX2319 inhibition of Escherichia coli AcrB multidrug efflux pump and comparison with other inhibitors. *Antimicrobial agents and chemotherapy* **58,** 6224–6234; 10.1128/AAC.03283-14 (2014).

38. Sjuts, H. *et al.* Molecular basis for inhibition of AcrB multidrug efflux pump by novel and powerful pyranopyridine derivatives. *Proceedings of the National Academy of Sciences of the United States of America* **113,** 3509–3514; 10.1073/pnas.1602472113 (2016).

39. Gohlke, H., Kiel, C. & Case, D. A. Insights into protein-protein binding by binding free energy calculation and free energy decomposition for the Ras-Raf and Ras-RalGDS complexes. *Journal of molecular biology* **330,** 891–913; 10.1016/s0022-2836(03)00610-7 (2003).

40. Wang, E. *et al.* End-Point Binding Free Energy Calculation with MM/PBSA and MM/GBSA: Strategies and Applications in Drug Design. *Chemical reviews* **119,** 9478–9508; 10.1021/acs.chemrev.9b00055 (2019).

41. Fairweather, S. J. *et al.* Coordination of Substrate Binding and Protonation in the N. gonorrhoeae MtrD Efflux Pump Controls the Functionally Rotating Transport Mechanism. *ACS infectious diseases* **7,** 1833–1847; 10.1021/acsinfecdis.1c00149 (2021).

42. Klenotic, P. A., Moseng, M. A., Morgan, C. E. & Yu, E. W. Structural and Functional Diversity of Resistance-Nodulation-Cell Division Transporters. *Chemical reviews* **121,** 5378–5416; 10.1021/acs.chemrev.0c00621 (2021).

43. Schott-Verdugo, S. & Gohlke, H. PACKMOL-Memgen: A Simple-To-Use, Generalized Workflow for Membrane-Protein-Lipid-Bilayer System Building. *Journal of chemical information and modeling* **59,** 2522–2528; 10.1021/acs.jcim.9b00269 (2019).

44. Tian, C. *et al.* ff19SB: Amino-Acid-Specific Protein Backbone Parameters Trained against Quantum Mechanics Energy Surfaces in Solution. *Journal of chemical theory and computation* **16,** 528–552; 10.1021/acs.jctc.9b00591 (2020).

45. Dickson, C. J., Walker, R. C. & Gould, I. R. Lipid21: Complex Lipid Membrane Simulations with AMBER. *Journal of chemical theory and computation* **18,** 1726–1736; 10.1021/acs.jctc.1c01217 (2022).

46. Izadi, S., Anandakrishnan, R. & Onufriev, A. V. Building Water Models: A Different Approach. *The journal of physical chemistry letters* **5,** 3863–3871; 10.1021/jz501780a (2014).

47. Joung, I. S. & Cheatham, T. E. Determination of alkali and halide monovalent ion parameters for use in explicitly solvated biomolecular simulations. *The journal of physical chemistry. B* **112,** 9020–9041; 10.1021/jp8001614 (2008).

48. Vargiu, A. V. *et al.* Computer simulations of the activity of RND efflux pumps. *Research in microbiology* **169,** 384–392; 10.1016/j.resmic.2017.12.001 (2018).

49. Reading, E. *et al.* Perturbed structural dynamics underlie inhibition and altered efflux of the multidrug resistance pump AcrB. *Nature communications* **11,** 5565; 10.1038/s41467-020-19397-2 (2020).
