# Supplementary material for: Conformational plasticity across phylogenetic clusters of RND multidrug efflux pumps and its impact on substrate specificity
Source: Nat Commun. 2025 Nov 26;16:11649. doi: 10.1038/s41467-025-66751-3 (PMC12749989; doi:10.1038/s41467-025-66751-3)
Supplement: Supplementary file 2 — Reporting Summary [file 41467_2025_66751_MOESM2_ESM.pdf]

nature portfolio

Corresponding author(s): Klaas M. Pos
Achilleas S. Frangakis,
Kay Diederichs
Satoshi Murakami

Last updated by author(s): Oct 30, 2025

Reporting Summary

Nature Portfolio wishes to improve the reproducibility of the work that we publish. This form provides structure for consistency and transparency in reporting. For further information on Nature Portfolio policies, see our Editorial Policies and the Editorial Policy Checklist.

Statistics

For all statistical analyses, confirm that the following items are present in the figure legend, table legend, main text, or Methods section.

n/a	Confirmed	
☐	☒	The exact sample size (n) for each experimental group/condition, given as a discrete number and unit of measurement
☐	☒	A statement on whether measurements were taken from distinct samples or whether the same sample was measured repeatedly
☒	☐	The statistical test(s) used AND whether they are one- or two-sided<br>*Only common tests should be described solely by name; describe more complex techniques in the Methods section.*
☒	☐	A description of all covariates tested
☐	☒	A description of any assumptions or corrections, such as tests of normality and adjustment for multiple comparisons
☐	☒	A full description of the statistical parameters including central tendency (e.g. means) or other basic estimates (e.g. regression coefficient) AND variation (e.g. standard deviation) or associated estimates of uncertainty (e.g. confidence intervals)
☒	☐	For null hypothesis testing, the test statistic (e.g. F, t, r) with confidence intervals, effect sizes, degrees of freedom and P value noted<br>*Give P values as exact values whenever suitable.*
☒	☐	For Bayesian analysis, information on the choice of priors and Markov chain Monte Carlo settings
☒	☐	For hierarchical and complex designs, identification of the appropriate level for tests and full reporting of outcomes
☒	☐	Estimates of effect sizes (e.g. Cohen's d, Pearson's r), indicating how they were calculated

Our web collection on statistics for biologists contains articles on many of the points above.

Software and code

Policy information about availability of computer code

Data collection	Cryo-EM data collection: Serial-EM v3.8 or EPU v2.12 (Thermo Scientific, Waltham, USA)
Data analysis	Cryo-EM data analysis: cryoSPARC v3.2 (Punjani et al. 2017), Relion v4.0 (Scheres 2012); X-ray data analysis: XDS (BUILT=20220110, Kabsch 2010), the Phenix package v.1.20.1 (Adams et al. 2010; Adams et al. 2002), MOLREP v11.0 (Vagin und Teplyakov 1997), Phaser v2.7 (McCoy et al. 2007); Model building, refinement and verification: Refmac v5 (Murshudov et al. 1997), Coot v0.9 (Emsley et al. 2010), MolProbity v4.02 (Chen et al. 2010); Ensemble docking: GNINA v1.3; homology modelling calculations: MODELLER v10.2; ligand protonatin states: ChemAxon Marvin suite; Binding free energy calculation: AMBER v22; Assignement GAFF2 atom types for ligands: AmberTools v23; Membrane embedding: PACKMOL-Memgen; multistep structural relaxation: AMBER v24; Structure visualisation: ChimeraX v1.6 (Pettersen et al. 2021):

For manuscripts utilizing custom algorithms or software that are central to the research but not yet described in published literature, software must be made available to editors and reviewers. We strongly encourage code deposition in a community repository (e.g. GitHub). See the Nature Portfolio guidelines for submitting code & software for further information.

Data

Policy information about availability of data

All manuscripts must include a data availability statement. This statement should provide the following information, where applicable:

- Accession codes, unique identifiers, or web links for publicly available datasets
- A description of any restrictions on data availability
- For clinical datasets or third party data, please ensure that the statement adheres to our policy

The crystallographic structures generated in this study have been deposited in the PDB database under following accession codes: AcrB V612W with bound minocycline: 9FE2 (https://dx.doi.org/10.2210/pdb9fe2/pdb), AcrB V612W apo: 9FE3 (https://dx.doi.org/10.2210/pdb9fe3/pdb), AcrB V612F with bound minocycline: 9FHC (https://dx.doi.org/10.2210/pdb9fhc/pdb), AcrB V612F, apo: 9FE4 (https://dx.doi.org/10.2210/pdb9fe4/pdb), AcrB V612N (TTT state): 9FHJ (https://dx.doi.org/10.2210/pdb9fhj/pdb), AcrB V612N (LTO state): 9FHG (https://dx.doi.org/10.2210/pdb9fhg/pdb), OqxB (TTO state): 8ZXS (https://dx.doi.org/10.2210/pdb8zxs/pdb). The cryo-EM structures generated in this study have been deposited in the PDB and EMDB database under following accession codes: OqxB in salipro nanodiscs: 9FDZ/EMD-50334 (https://dx.doi.org/10.2210/pdb9fdz/pdb), OqxB monomer classes: EMD-50335, AcrB V612F monomer in the O state: 9FDQ/EMD-50332 (https://dx.doi.org/10.2210/pdb9fdq/pdb), AcrB V612W monomer in the O state: 9FDP/EMD-50331 (https://dx.doi.org/10.2210/pdb9fdp/pdb), AcrB wildtype in DDM: EMD-50328, AcrB V612F in DDM: EMD-50329, AcrB wildtype in salipro nanodiscs: EMD-50645. Single particle cryo-EM maps (.mrc format) as shown in the Supplementary Figures are available for download under https://doi.org/10.6084/m9.figshare.28255283. For reference and comparison, the previously published structures available in the PDB database under following accession codes have been used: 4dx5 (https://dx.doi.org/10.2210/pdb4dx5/pdb), 1iwg (https://dx.doi.org/10.2210/pdb1iwg/pdb) , 7cz9 (https://dx.doi.org/10.2210/pdb7cz9/pdb), 5o66 (https://dx.doi.org/10.2210/pdb5o66/pdb), 6zoe (https://dx.doi.org/10.2210/pdb6zoe/pdb), 7wls (https://dx.doi.org/10.2210/pdb7wls/pdb), 7kgh (https://dx.doi.org/10.2210/pdb7kgh/pdb), 6ta6 (https://dx.doi.org/10.2210/pdb6ta6/pdb), 6t7s (https://dx.doi.org/10.2210/pdb6t7s/pdb), 5lq3 (https://dx.doi.org/10.2210/pdb5lq3/pdb). For reference for model building the predicted structure available in the AlphaFold Structure Database under UniProt accession number U5U6L7 was used. The PDB files of the top docking poses with chloramphenicol, minocycline, doxorubicin, and erythromycin on the E. coli efflux pump AcrB wildtype, V612F and V612W variants have been deposited in the zenodo database under following accession code: 10.5281/zenodo.14719971. Molecular dynamics simulation trajectories of chloramphenicol and minocycline in complex with wildtype AcrB and the V612F and V612W variants have been deposited in the zenodo database under following accession code 10.5281/zenodo.15489434. Source data and raw data for the plate dilution assay, MIC determination and the accumulation assay are available as additional source and raw data files. Unless otherwise stated, all data supporting the results of this study can be found in the article, supplementary, and source data files. Source Data are provided with this paper.

Research involving human participants, their data, or biological material

Policy information about studies with human participants or human data. See also policy information about sex, gender (identity/presentation), and sexual orientation and race, ethnicity and racism.

Reporting on sex and gender	This study does not include any human participants, their data or their biological material
Reporting on race, ethnicity, or other socially relevant groupings	This study does not include any human participants, their data or their biological material
Population characteristics	This study does not include any human participants, their data or their biological material
Recruitment	This study does not include any human participants, their data or their biological material
Ethics oversight	This study does not include any human participants, their data or their biological material

Note that full information on the approval of the study protocol must also be provided in the manuscript.

Field-specific reporting

Please select the one below that is the best fit for your research. If you are not sure, read the appropriate sections before making your selection.

☒ Life sciences ☐ Behavioural & social sciences ☐ Ecological, evolutionary & environmental sciences

For a reference copy of the document with all sections, see nature.com/documents/nr-reporting-summary-flat.pdf

Life sciences study design

All studies must disclose on these points even when the disclosure is negative.

Sample size	Sample size of the phylogenetic analysis was based on the representative proteins of the HAE-1 RND transporter family in the transporter classification database (Saier et al. 2006) (accessed 18.08.2023) with addition of the BpeF and CmeB sequences.<br>Minimum of three biological replicates were tested for all functional assays. N=3 was considered sufficient since the results were reproducible and it is usual in the field.<br>X-ray structures were derived from diffraction data for one crystal per sample as is usual in the field.<br>Cryo-EM data were derived from one grid per sample since it provided a sufficient amount of good particles to determined 3D density maps with sufficient resolution.

Data exclusions	No data were excluded.
Replication	Biological and technical replicates were performed to asses the reproducibility of the results. All findings were reproducible.
Randomization	For functional assays, clones were picked randomly after transformation in E. coli cells. For protein level structural characterisation, randomisation is not applicable.
Blinding	For functional assays, clones were picked randomly after transformation in E. coli cells. For protein level structural characterisation, blinding is not applicable.

Reporting for specific materials, systems and methods

We require information from authors about some types of materials, experimental systems and methods used in many studies. Here, indicate whether each material, system or method listed is relevant to your study. If you are not sure if a list item applies to your research, read the appropriate section before selecting a response.

Materials & experimental systems

n/a	Involved in the study	
☐	☒	Antibodies
☒	☐	Eukaryotic cell lines
☒	☐	Palaeontology and archaeology
☒	☐	Animals and other organisms
☒	☐	Clinical data
☒	☐	Dual use research of concern
☒	☐	Plants

Methods

n/a	Involved in the study	
☒	☐	ChIP-seq
☒	☐	Flow cytometry
☒	☐	MRI-based neuroimaging

Antibodies

Antibodies used	primary rabbit anti-AcrB antibody (dilution of 1:10,000; Neosystems, France, custom-antibody) and then, with a secondary goat anti-rabbit IgG (whole molecule)-alkaline phosphatase antibody (dilution of 1:1,500; A3687, Sigma-Aldrich, St. Louis, USA).
Validation	All the commercial antibodies were verified by the manufacturers according to immunoblots and/or image on their websites (Sigma-Aldrich refers for Western Blot analysis validation to Cibelli et al., 2001 (PMID: 11298794 DOI: 10.1046/j.0953-816x.2001.01510.x).. Anti-AcrB has been validated in our lab with purified AcrB as sample. e.g. Tam, H.-K. et al. Allosteric drug transport mechanism of multidrug transporter AcrB. Nature communications 12, 3889; 10.1038/s41467-021-24151-3 (2021) or Seeger MA, et al. (2008) Engineered disulfide bonds support the functional rotation mechanism of multidrug efflux pump AcrB. Nat Struct Mol Biol 15:199–205).

Plants

Seed stocks	Plants were not used in this study.
Novel plant genotypes	Plants were not used in this study.
Authentication	Plants were not used in this study.
